# A Functional Magnetic Resonance Imaging Assessment of Small Animals’ Phobia Using Virtual Reality as a Stimulus

**DOI:** 10.2196/games.2836

**Published:** 2014-06-27

**Authors:** Miriam Clemente, Beatriz Rey, Aina Rodriguez-Pujadas, Juani Breton-Lopez, Alfonso Barros-Loscertales, Rosa M Baños, Cristina Botella, Mariano Alcañiz, Cesar Avila

**Affiliations:** ^1^LabHumanI3BHUniversitat Politecnica de ValenciaValenciaSpain; ^2^Ciber, Fisiopatología Obesidad y Nutrición, CB06/03Instituto de Salud Carlos IIIMadridSpain; ^3^Departamento de Psicología Básica, Clínica y PsicobiologíaUniversitat Jaume ICastellonSpain; ^4^LabpsitecUniversitat Jaume ICastellonSpain; ^5^LabpsitecUniversitat de ValenciaValenciaSpain

**Keywords:** neuroimaging, patient assessment, virtual reality, phobia

## Abstract

**Background:**

To date, still images or videos of real animals have been used in functional magnetic resonance imaging protocols to evaluate the brain activations associated with small animals’ phobia.

**Objective:**

The objective of our study was to evaluate the brain activations associated with small animals’ phobia through the use of virtual environments. This context will have the added benefit of allowing the subject to move and interact with the environment, giving the subject the illusion of being there.

**Methods:**

We have analyzed the brain activation in a group of phobic people while they navigated in a virtual environment that included the small animals that were the object of their phobia.

**Results:**

We have found brain activation mainly in the left occipital inferior lobe (*P*<.05 corrected, cluster size=36), related to the enhanced visual attention to the phobic stimuli; and in the superior frontal gyrus (*P*<.005 uncorrected, cluster size=13), which is an area that has been previously related to the feeling of self-awareness.

**Conclusions:**

In our opinion, these results demonstrate that virtual stimulus can enhance brain activations consistent with previous studies with still images, but in an environment closer to the real situation the subject would face in their daily lives.

##  Introduction

### Small Animal Phobia

Phobias are one of the most wide spread and common disorders of modern life, affecting 1 in 10 people at some point of their lives [1-3]. More specifically, small animals’ phobia is one of the most disabling ones, due to the possibility of facing the animal in daily life. In fact, 40% of specific phobias belong to the category of small animals, including bugs, mice, snakes, and bats [4].

### Evaluating State of the Phobia

In order to evaluate the state and evolution of the phobia, many studies have been conducted using brain imaging techniques, such as functional magnetic resonance imaging (fMRI), positron emission tomography (PET), or electroencephalography (EEG). To date, most of those studies used photographs or videos of real animals as stimulus to provoke the reaction of the subject. For example, Paquette et al [5] used film excerpts of real spiders as stimulus, and excerpts of real butterflies as control. They aimed to probe the effects of cognitive behavioral therapy (CBT) on the neural correlates of spider phobia. They found significant activation of the right dorsolateral prefrontal cortex and the parahippocampal gyrus during the phobogenic stimulus before the CBT, which disappeared after it.

Regarding the studies that used pictures as stimulus, several of them analyzed the amygdala activation when comparing phobic and nonphobic subjects. For example, Schienle et al [6] studied the effects of a cognitive therapy, comparing pictures of real spiders with images that provoked fear, disgust, or images that were neutral. Another example is the study of Larson et al [7], who also used pictures as stimulus. Alpers et al [8] studied the amygdala activation and its relationship with attention over spider-phobic women, employing superimposed images of spiders and birds during the task. Apart from the amygdala, several studies that used pictures as stimuli were focused on other brain areas, such as the insula. Straube et al [9] analyzed insula activation due to the anticipatory anxiety, which refers to the fear you feel when you are expecting to find the animal object of your phobia. In another study, Wendt et al [10] analyzed the relation of the insula activation to the defensive response the subject experiences before the appearance of their feared object, using sustained exposure to the phobia relevant stimuli. Regarding other neuroimaging techniques, Scharmüller et al [11] used EEG to investigate the symptoms of spider phobia, again using pictures as stimulus. They found sources of activation in areas related to the visuo-attentional processing, emotional processing, and representations of aversive bodily states.

### Virtual Reality Benefits

In the field of phobias, virtual reality (VR) has been repeatedly used to treat the disorder, but to our knowledge, it has not been used for the assessment of the disturbance yet. VR is a technology that comprises computer-generated simulations of reality [12]. Among most common treatments for mental disorders are the cognitive behavioral treatments, based on the exposure of the subject to the object of their fear, to make them adapt progressively to the stimulus [13,14]. However, these direct-exposing techniques sometimes are considered “dangerous and ethically reprehensible” [14-16] because of the impact that the direct exposure can have on the subject. In this sense, VR allows exposure of the subject to the feared stimuli in a controlled environment that is considered safer and more ethically acceptable [17]. Botella et al [18] gave a list of the advantages that VR has in psychotherapy; from which we can emphasize the aforementioned allowance of running the therapy in a protected environment close to reality, where the patient can act without feeling threatened (from a “safe base”). Furthermore, in VR the patient can interact with the context, and the psychotherapist can grade the situation according to the patient’s state. Moreover, in a more technical way, VR is an excellent source of information in performance achievements, and allows for an accurate control of the situation. Finally, we can mention the ecological validity of the stimuli presentation.

One of VR’s many advantages is that it allows the patient to interact with the phobic object or situation as if they are real and they exist there with the feared animals. This concept is known as presence, the sense of being there, inside the virtual environment, although your body is physically located elsewhere [19-22].

The VR exposure therapy has been widely used in the treatment of specific phobias [23], such as acrophobia [24], claustrophobia [25], arachnophobia [26], hodophobia [27], and pteromerhanophobia [28]. An example of this kind of treatment in our field of interest (small animals phobia) is the one developed by Garcia-Palacios et al [29], using VR with spider phobic subjects. In another study, Botella et al [30] applied augmented reality, a VR-related technology, for treating cockroach phobic subjects. However, to date, VR has not been used inside the fMRI scanner as a stimulus to assess the responses of the phobic subjects in the presence of the feared elements.

Our proposal is that the use of VR as a stimulus inside an fMRI setting will entail the same advantages to the phobia evaluation that it brought to the phobia treatment. Traditionally, the phobia evaluation has been made using behavioral assessments and self-reported scales. Finding the neural correlates related to the phobia activation in each individual subject could help in their evaluation process, grading their level of phobia, and giving further information about their state. This knowledge can help the therapists to decide the most appropriate treatment for each particular patient, taking into account the brain activations that can be observed. Besides, the analysis of the brain activations related to the phobia once the treatment has finished can be used to evaluate if the treatment has been successful. Using VR inside an fMRI setting will allow the psychologists to place the patients in virtual situations related to the object of their phobia, where they are able to interact. It can also make the user feel present in the environment, creating a more “realistic” experience than the one induced by the visualization of videos or photographs. Consequently, we expect the activated brain areas to be more similar to those activated during the real experience. Furthermore, the experimenter will have the possibility of controlling the exposure to the virtual situations in the most convenient way, customizing it according to the patient’s situation, if it is required. Finally, this kind of system allows an easy monitoring of the behavioral responses of the participants inside the virtual environment.

In order to validate this proposal, our target in the present work is to examine whether VR can be used for the assessment of the phobia, provoking a more realistic and immersive situation than the view of a still photograph, which can be manipulated by the psychologist. We have used virtual environments where the subject can navigate freely, which should induce a higher sense of presence due to the self-control of the navigation route [31]. As presented previously in Clemente et al [32], our main hypothesis is that the brain areas activated with these environments will be coherent with the results from previous studies based on pictures or videos of real animals (see Multimedia Appendix 1).

## Methods

### Recruitment

For this study, we recruited 11 right-handed phobic women, between 20 and 35 years old (mean age 24.64). None of them had any other medical or psychological disorders, apart from the phobia. The participants’ hand dominance was tested using the Edinburgh Handedness Inventory [33].

Expert clinicians who were also the therapists for the participants carried out the diagnosis and assessment phases. In order to be included in the study, the following inclusion criteria were considered: (1) meeting Diagnostic and Statistical Manual of Mental Disorders - 4^th^ edition (DSM-IV) [1] criteria for specific phobia animal type, specifically cockroach and spider phobia; (2) having scores over 4 in phobic avoidance (on a scale of 0 to 8); (3) having no current alcohol or drug dependency; (4) having no diagnosis of major depression or psychosis; (5) not having been or being treated with a similar program; and (6) having a minimum of 1 year of duration for the problem. The Anxiety Disorders Interview Schedule [34] specific phobia section was used to carry out the differential diagnosis of the anxiety disorders included in the DSM.

These women were students, were paid for their participation in the study, and were recruited from the Universitat Jaume I in Castellón. Ethical approval was obtained from the authors’ institution, and each subject signed a written informed consent prior to participation.

### Environments

**Figure 1:**
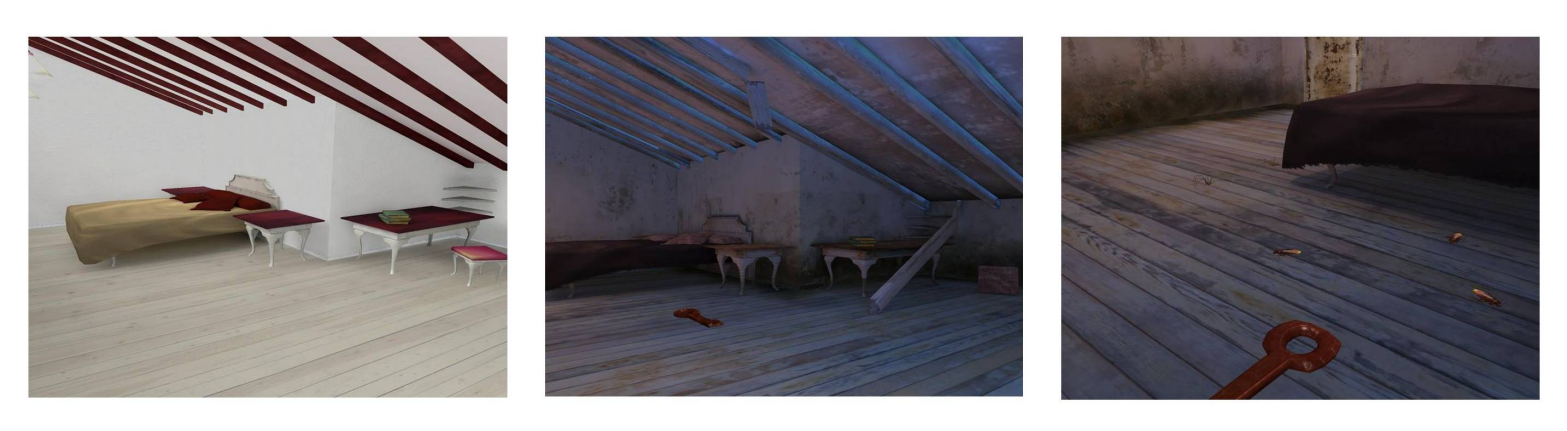
Captures of the three environments used in the conditions: CLEAN (left), DIRTY (center), and PHOBIC (right).

The virtual environments used during the task were programmed using GameStudio software (Conitec Datensysteme GmbH), which allowed us to develop three-dimensional objects and virtual worlds with which participants could interact and navigate. For this study, we have divided the task into three experimental conditions, all of them involving a room where the subject can navigate freely. In the first of these conditions “CLEAN”, the patient navigates through a common clean bedroom (with a bed, a closet, and a desk with some books on it). In the second condition “DIRTY”, the navigation is performed through the same room, but this time dirty and darker, giving the subject the feeling that the feared animal could appear in any moment; this room intends to stimulate the anxiety in the user. In the last condition “PHOBIC”, the subject navigates through the same dirty room, but this time there appeared spiders and cockroaches. Each of these experimental conditions lasted 20 seconds. Figure 1 shows the captures of each environment.

To prevent the subjects from staying still in the VR during the navigation periods, a search task was included in order to force them to move through the environment and confront the phobic stimulus in the correspondent experimental condition. This task consisted of searching and counting the number of red keys that appeared and disappeared in the environment. However, they were not encouraged to find them all, or to find them as quickly as possible, they were only told to continue searching for them during each period. After each task, the subjects were questioned about the number of keys that they had found (they had to answer in a short period of 4 seconds). While they were conducting the tasks, the researcher checked in the computer to see that they had answered properly. The number of keys that they counted was not relevant; it was just included to avoid the subjects remaining still during the experimental conditions.

**Figure 2:**
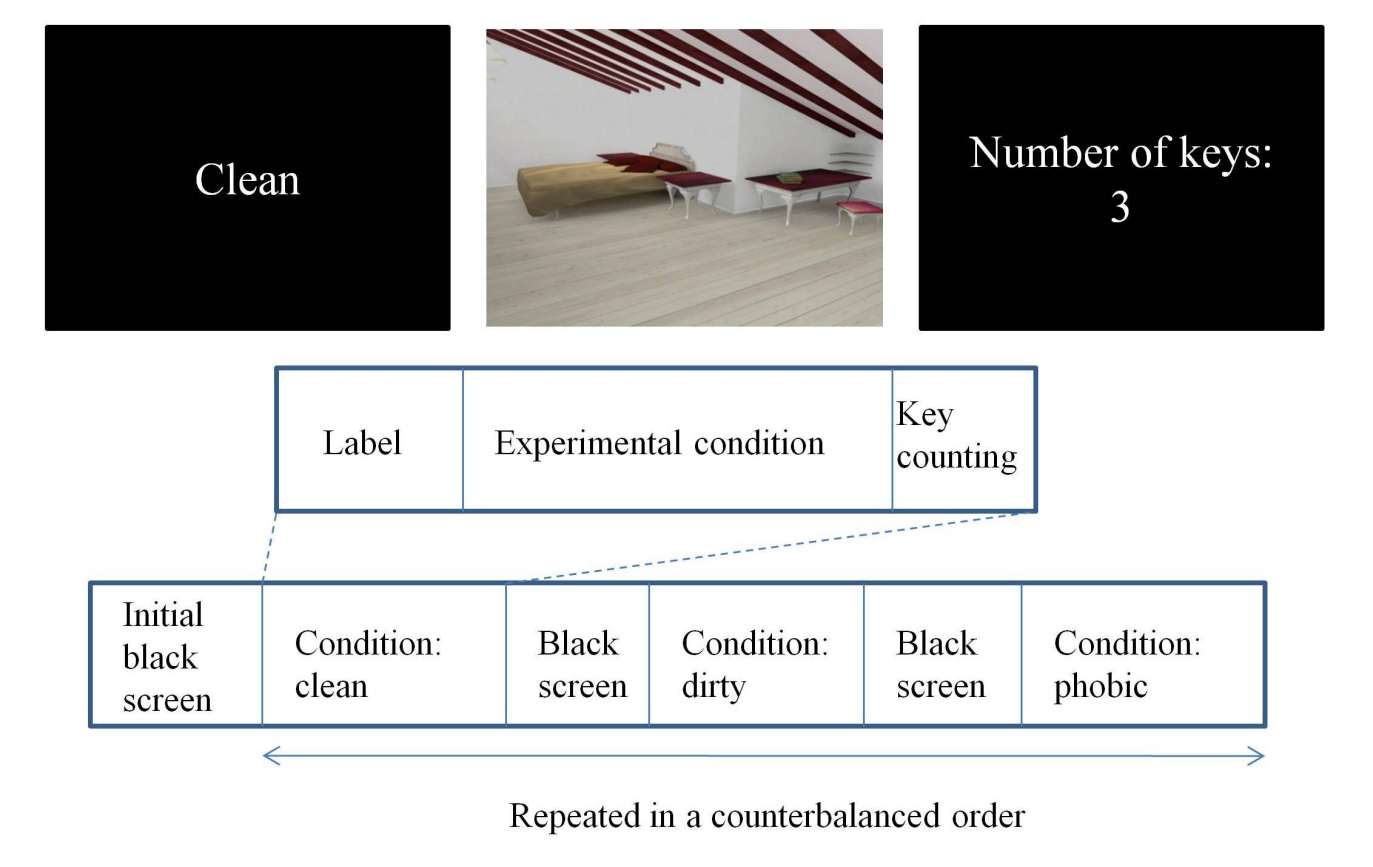
Scheme of the protocol for the functional magnetic resonance imaging (fMRI) task.

A black screen was included between phases to give the subjects a rest period, during which brain activation could decay to its baseline values (6 seconds). After this, the label indicating the next task appeared (2 seconds). The total time between tasks was 12 seconds. Each of the three experimental conditions was repeated six times in a counterbalanced order to prevent effects produced by the order in which they were presented. At the beginning of the experiment there were 14 seconds of black screen to compensate for relaxation time (T1) saturation effects. The total time of the complete experiment was 9 minutes and 40 seconds. Figure 2 shows a scheme of the protocol.

Before entering the scanner, the subjects underwent a short training session where they were introduced to the VR navigation task. This training session was conducted in a supplementary virtual environment, without any kind of phobic stimuli, to avoid habituation. During the fMRI scan, the VR application checked the total time that they spent moving the joystick in each condition to guarantee that they did not remain still during the phobic stimulation.

### Functional Magnetic Resonance Imaging Procedures

All the subjects were scanned in a 1.5 Tesla Siemens Avanto Magnetic Resonance scanning device. We used an adapted magnetic resonance (MR) helmet to prevent head movement. To display the environments, we used magnetic resonance imaging (MRI)-compatible video goggles, VisualStim Digital (Resonance Technology Inc), and for the navigation we used an adapted joystick (Resonance Technology Inc). First, sagittal T1-weighted structural images were acquired (224 x 256 matrix covering the brain with 176 contiguous 1 mm slices, repetition time [TR] = 11 ms, echo time [TE] = 4.94 ms, flip angle [FA] = 15⁰, voxel size = 1.04 x 1.04 mm). Then, the functional scanning was launched, synchronized with the virtual environments. Functional images were obtained using a relaxation time (T2*) single-shot echo-planar imaging sequence. We used 30 contiguous 4.2 mm interleaved axial slices—parallel to the anterior commissure – posterior commissure line (AC-PC line)—covering the entire volume of the brain with a 64 x 64 matrix (TR = 2000 ms, TE = 30 ms, FA = 90⁰, voxel size = 3.5 x 3.5 mm).

### Data Analysis

We have used the statistical parametric mapping software (SPM8, Wellcome Department of Imaging Neuroscience) for the analysis of the fMRI data, launched with the 7.1 version of Matlab (MathWorks). We excluded the first 7 scans from the analysis to eliminate the decay of the fMRI signal that is associated with the moment when magnetization reaches equilibrium. First we aligned the images to the AC-PC line. Once it was done, we began the preprocessing of the data, realigning the functional images (estimate and reslice option), coregistering them to the structural images, and segmenting this latter anatomical scan. We then normalized the resliced functional volumes with the normalization parameters that we extracted after segmentation and normalization of the anatomical volumes for each subject separately (the template was provided by the Montreal Neurological Institute, MNI). None of the volunteers had to be excluded due to movements or distortions during the fMRI. Finally, we smoothed the images using a Gaussian kernel (full width at half maximum, FWHM, of 8 x 8 x 8 mm).

In a first fixed-effect level analysis, the functional time series for each subject and for each condition were modeled with a boxcar function convolved with the hemodynamic response function. The parameters for the motion correction were employed as regressors of noninterest. To eliminate the low frequency components in the signal caused by the scanner motion and warming, we applied a 96 seconds high pass filter.

We performed group tests at a second level random effect analysis. We tested for task related activation by performing a one-sample *t* test, including contrast images of estimated parameters for the differences of interest between conditions. As we have aforementioned, our fMRI paradigm was divided into three different navigation tasks (clean room, dirty room, and phobic-stimulus room) that we wanted to compare in order to obtain the contrasting brain activations. Although the results for the three contrasts have been obtained, the results that show the brain activations for the phobic stimulus are contained in the “phobic>clean” contrast. The “phobic>dirty” contrast shows phobic activations avoiding the anxiety feeling caused by the dirtiness of the room, and the “dirty>clean” contrast contains the anxiety related activations.

All contrasts at group level were considered if more than 10 adjacent voxels passed the statistical threshold of *P*<.005 (uncorrected). These results were corrected at *P*<.05 using AlphaSim correction (combined height threshold *P*<.005, and a minimum cluster size= 25) [35]. We used the xjView software utility for statistical parametric mapping (SPM) which uses the MNI coordinates system to obtain the specific brain areas that were activated for each contrast.

## Results

**Figure 3:**
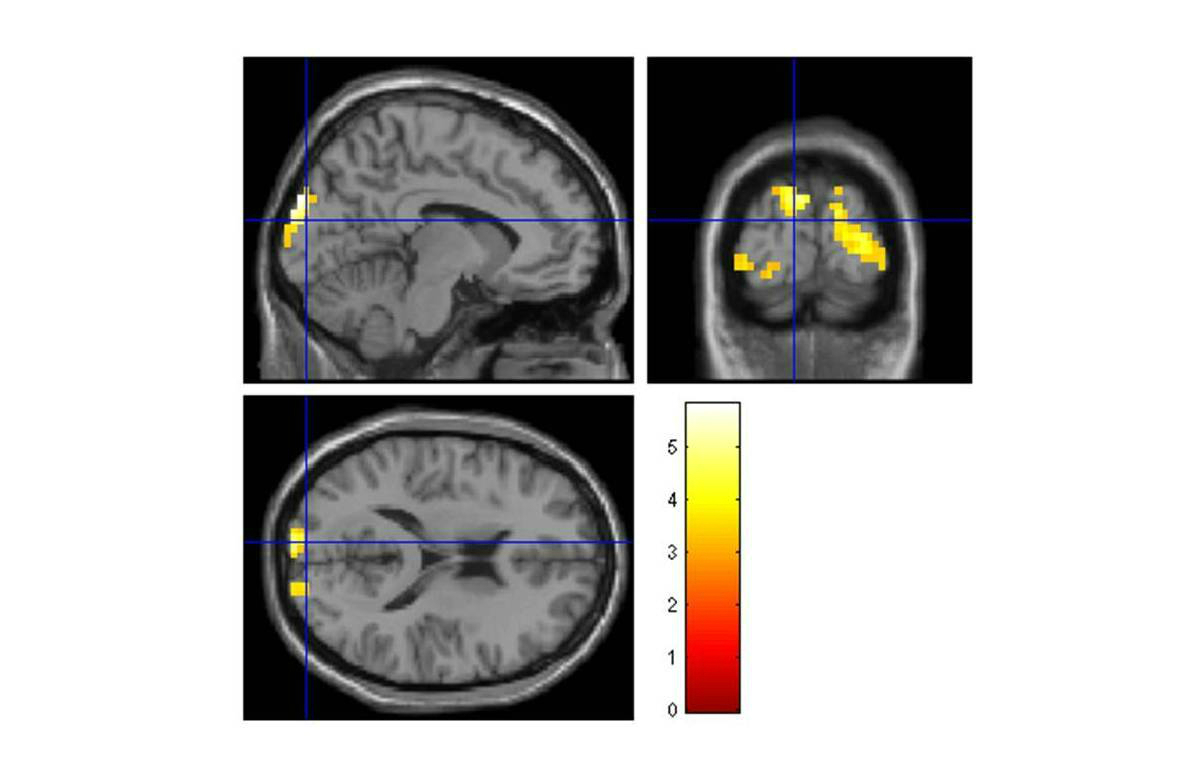
Brain activations for the “phobic>clean” contrast.

**Figure 4:**
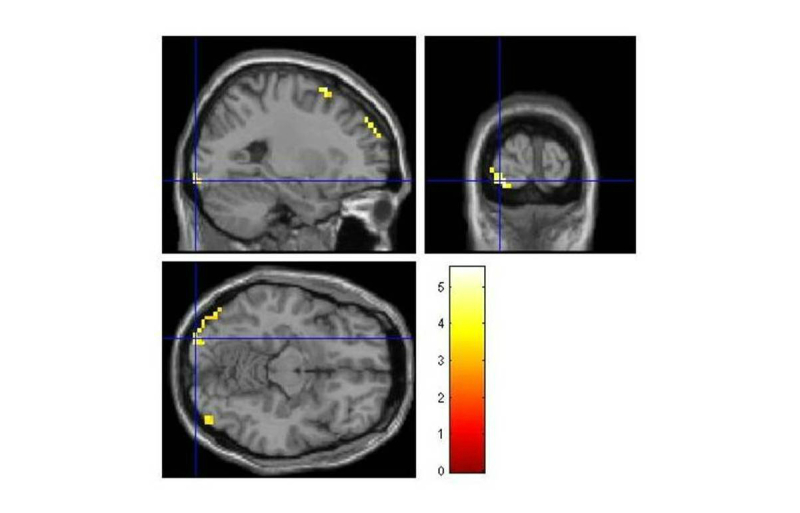
Brain activations for the “phobic>dirty” contrast.

**Figure 5:**
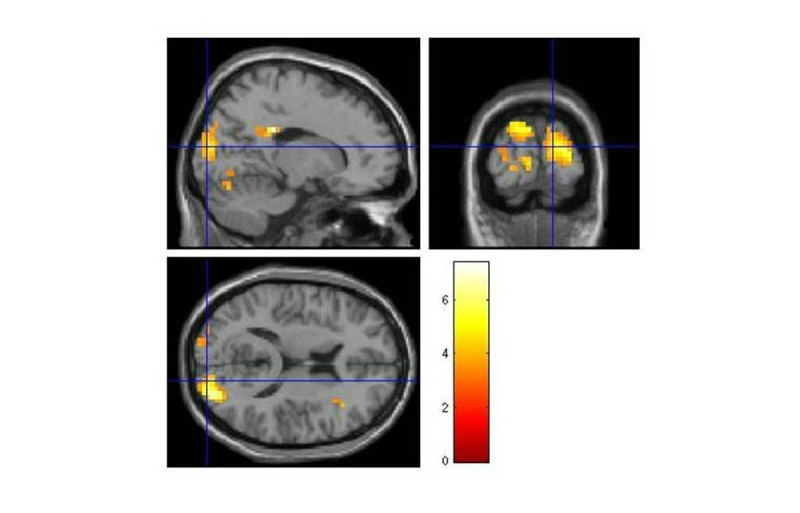
Brain activations for the “dirty>clean” contrast.

We selected the contrast “phobic>clean”, and looked for the main activated brain regions. We found activations in the left occipital inferior lobe and middle occipital gyrus bilaterally, among others (see “superior” part of Table 1, and Figure 3 also shows this activation). Brain regions that also displayed significant activations during the task were: (1) the cuneus bilaterally, (2) the superior frontal gyrus, and (3) the precuneus. In the middle part of Table 1 (also shown in Figure 4), we can observe the results obtained for the “phobic>dirty” contrast (inferior occipital lobe bilaterally, and the left superior, and middle frontal lobe); and in the lower part of Table 1 (also shown in Figure 5) are the results for the “dirty>clean” contrast (left superior occipital lobe, and right middle frontal gyrus, middle occipital gyrus and cingulate). In the table, apart from the anatomical area and brain hemisphere, the values for the location and T score of the maximum for each area, the size of the cluster activated in each area, and the *P* value used as threshold are shown.

**Table 1:** Brain area activation results.

Contrast	Anatomical region	Hemisphere	(x, y, z)	*t*_10_	Cluster size	*P*
“Phobic>clean”	Occipital inferior lobe	L^a^	(-22, 98, -12)	4,19	36	<.05 corrected
	Middle occipital gyrus (BA19^c^)	L^a^	(-54,-77, -4)	5,21	29	<.05 corrected
	Middle occipital gyrus	R^b^	(31, 77, 0)	4,76	175	<.05 corrected
	Cuneus	R^b^	(20, -91, 9)	4,01	36	<.05 corrected
	BA18^c^	R^b^	(26, -96, 6)	-11,64	28	<.05 corrected
	Cuneus	L^a^	(-8, -95, 30)	5,82	55	<.05 corrected
	Superior frontal gyrus	R^b^	(20, 49, 42)	4,52	13	<.005 uncorrected
	Precuneus	L^a^	(-1, -46, 68)	4,59	31	<.05 corrected
“Phobic>dirty”	Inferior occipital lobe	L^a^	(-26, -98, -12)	5,52	54	<.05 corrected
	Inferior occipital lobe	R^b^	(48, -84, -8)	4,43	22	<.005 uncorrected
	Superior frontal lobe	L^a^	(-22, 56, 34)	4,51	18	<.005 uncorrected
	Middle frontal lobe	L^a^	(-26, 14, 63)	5,25	18	<.005 uncorrected
“Dirty>clean”	Superior occipital lobe	L^a^	(-15, -91, 30)	5,69	201	<.05 corrected
	Middle frontal gyrus	R^b^	(24, 53, -8)	5,23	39	<.05 corrected
	Middle occipital gyrus	R^b^	(27, -84, 13)	6,81	184	<.05 corrected
	Cingulate gyrus	R^b^	(17, -35, 30)	7,40	14	<.005 uncorrected

^a^L=left

^b^R=right

^c^BA = Brodmann area

##  Discussion

### Main Goal

The main goal of our study was to analyze the brain areas activated due to phobic stimulus during a subject’s navigation in a virtual environment through the three experimental conditions previously described (CLEAN, DIRTY, and PHOBIC). The main results, for the purposes of the study, are those obtained when comparing the brain activations between the phobic and the clean conditions (“phobic>clean”), which reflect the fear and anxiety felt by the subjects due to the phobic stimulus when compared with an emotionally neutral situation. Both the phobic and dirty situations may generate anxiety in the participant. However, in the dirty condition, the anxiety is generated by the fact of being in a threatening room (because of the dirtiness of the room, the participant may feel that is a dangerous place to be), and in the phobic condition, apart from the dirtiness of the room there are phobic stimuli. Our hypothesis is that in the “phobic>clean” comparison, the activations may be caused by both factors, while in the “phobic>dirty” contrast, the activations would be related to the phobia itself, and not to the anxiety feeling.

In the following paragraphs, we will comment on the results of the “phobic>clean” contrast in comparison with the results obtained in other studies about phobia. After that, we will discuss briefly the results obtained in the other two contrasts, “phobic>dirty” and “dirty>clean”. Finally, the limitations of the study will be commented on, and we will make some overall conclusions.

### Principal Results

One of the most important activated areas in the “phobic>clean” contrast is the occipital lobe, more specifically, the left inferior area and in the middle lobe bilaterally. Another important result was the activation obtained in the superior frontal gyrus. Finally, activations were also found in the cuneus and precuneus. A more detailed explanation of the importance of these results is found below.

### The Occipital Lobe

The occipital lobe mainly controls the visual areas, which are necessary for the performance of a navigation task. In the inferior area of the occipital lobe, we found activation in the lingual gyrus, which is believed to play a role in dreaming as well as in vision, especially in the recognition of words [36]. In this area, we also found activation in the Brodmann area 18, part of the extrastriate visual cortex. This encompasses multiple functional areas, including V3, V4, V5/MT, which are sensitive to motion, or the extrastriate body area used in the perception of human bodies [37,38].

Paquette et al had already found brain activation in the occipital lobe area [5] in a similar study carried out using film excerpts of spiders as the phobic stimulus, and film excerpts of butterflies as the neutral condition. They concluded that this activation was related to enhanced visual attention to the phobic stimuli, and that it supported vigilance functions in anxiety [39,40]. Moreover, those results were consistent with those obtained in other similar studies [41-43]. More recently, there have been several fMRI [6,8,9] and PET [11] studies among phobic and nonphobic subjects that have also found activation in the visual cortex. In fact, Straube et al [9] justified it as likely to be caused by the attention subjects put on the visual input that reflect an “increase in the processing of the cue, but also the expectation of behaviorally relevant sensory input”. Moreover, several studies have pointed out the spread over time that the amygdala activation has to the occipital areas, due to the emotional relevance of the stimulus [44,45].

### The Superior Frontal Gyrus

The other important activation in our results was found in the superior frontal gyrus. This area has been previously related to the feeling of self-awareness [46], which is increased when the phobic subjects watch the animal that provokes their fear. During the resting condition (CLEAN), the subjects are involved in a highly demanding sensory task during which they relax and are less conscious of themselves [46]. However, when the environment changes, and the phobic subjects find themselves in a fearful situation, their alert state increases, trying to inhibit their reaction in front of the phobic stimulus [5]. The natural reply to this stimulus is to avoid the fear response it provokes over them, and to do so they control their mind and body, increasing the consciousness they have of themselves. Also according to Du Boisgueheneuc et al [47], the superior frontal gyrus is related to the higher cognitive functions and the working memory.

However, there are some other studies that relate the visualization of a phobic stimulus to the deactivation of the frontal areas, especially of the prefrontal and orbitofrontal cortices [48,49]. They argue that visually elicited phobic reactions deactivate the areas related to cognitive control over emotions, resulting in motor readiness to fight or flight [49]. Nevertheless, those deactivations are related to more anterior areas of the brain, not involving the superior frontal gyrus. Moreover, in our study the subjects were mild phobics, so they were able to react in front of a phobic stimulus without needing to engage more instinctive reactions.

Although Paquette et al [5] did not find activation in the superior frontal gyrus, they discussed its relation to the voluntary self-regulation of emotion. More exactly, they exposed the results obtained in a PET study carried out by Johanson et al [50], where an increase in the frontal regional cerebral blood flow was obtained which correlated with the use of cognitive strategies to cope with the phobic situation. Paquette et al [5] pointed out that the phobic subjects activated their prefrontal areas when attempting to control their fear before the film excerpts of spiders. Goldberg et al [46] analyzed the subjective awareness feeling, and its relation with the frontal areas of the brain. They remarked how when watching an absorbing movie or being involved in a highly demanding sensory task (as in our case the virtual navigation through an immersive environment), the strong subjective feeling is of “losing the self”, or as they explained, of disengaging from self-related reflective processes. Accepting this state, the increase in the self-awareness feeling during the visualization of phobic stimulus in a highly demanding navigation task is clearly related to the higher feeling of yourself when “fighting” the fear. In the words of Scharmüller et al [11], increased activation in the superior frontal cortex might reflect the patients’ urge to flee during the confrontation with the feared object; and this link between the sensorimotor system and the effective/cognitive function is in line with the theory about embodied cognition [51]. In conclusion, we can consider this activation essentially related to the phobia.

### The Cuneus and the Precuneus

With respect to our other results, we also found activity in the cuneus and the precuneus. Regarding the cuneus, it has been related to visual processing, which is directly associated with the sense of presence that the subject feels while navigating through a virtual environment [52]. On the other side, the precuneus has been related to self-consciousness, such as reflective self-awareness, that involves rating your own personality traits [53,54]. This information continues with the idea of an increase in the consciousness of yourself while you are exposed to a phobic stimulus, trying to reduce your reaction before it. It has also been involved in directing attention in space when planning or performing a movement [55,56], which is directly related to the navigation through a virtual environment.

### The Amygdala

Although one of the areas most commonly related to phobias is the amygdala, it is not activated in our results. Several previous studies have been conducted to find the pattern of activation of this area [5,7,8], they have concluded that the amygdala suffers habituation over time [7]. This means that it is activated for a brief time period and then disappears. Paquette et al [5] also pointed out that this suggests that the amygdala may not be related to the phobic expression or experience, but to the fear conditioning [5,57]. Straube et al [9] also discussed that the amygdala activation may occur during brief presentations of the phobogenic stimuli, and in the induction of rapid behavioral responses, more than in the sustained and explicit processing of the threatening stimuli. Alpers et al [8] also pointed out that their activation in the amygdala was helped by the brief stimulus they used (200 ms). In our case, the use of periods of navigation as stimulus instead of pictures may be the cause of not detecting activation in this area (we used a block design for the protocol instead of an event-related). In fact, most of the studies around the amygdala have reported its activation during the very early stages of the stimulus [6-8].

### The “Phobic>Dirty” Contrast

Having exposed the main results for the “phobic>clean” contrast, we will briefly discuss the results for the remaining contrasts. Regarding the “phobic>dirty” comparison, we found that the inferior occipital lobe played a major role in the fear response to the phobic stimulus, bilaterally. This is in concordance with the results obtained for the “phobic>clean” contrast, where we pointed out the relation of this area with the phobic response. As we have already said, the occipital lobe has been related to enhanced visual attention to the phobic stimuli [39,40]. The other important activation was located in the superior and middle frontal lobe, the result also contained in our previous contrast, due to its relation to the feeling of self-awareness and the action of the sensory system [46]. As we can see, the main results that we pointed out as being related to the phobia are still activated when we restrict the conditions of the contrast to avoid the anxiety results.

### The “Dirty>Clean” Contrast

Regarding the “dirty>clean” contrast, the self-awareness was still high due to the greater fear of finding a spider or a cockroach when navigating through a dark and dirty environment than when navigating through a clean one, which resulted in the activation of the middle frontal gyrus. The activation of the occipital lobe was maintained here due to the higher visual processing when expecting the appearance of a feared animal. The last activation was located in the cingulate gyrus, which Paquette et al [5] pointed out to be mainly associated with the cognitive/internal generation of the emotional state by evoking visual imagery or memories. As we aforementioned, the activations in this contrast are due to the evocation of the fear, not to the exposition to it; so the meaning of the activation in the cingulate gyrus is clear as a generator of the emotional evocations.

### Limitations

To finish this discussion, we will address some of the limitations that our study presents. First of all, it was conducted using a specific group of participants, namely, 11 right-handed women. This constitutes a small sample size, which restricts the statistical power of the study to detect changes in the blood-oxygen-level-dependent signal. All of the subjects were right-handed in order to prevent noise effects due to manual lateralization on brain activation in the virtual/spatial processing. Moreover, we chose all women in order to reduce the variability generated by gender differences. In fact, some previous studies have pointed out the importance of choosing only women, due to their higher activation in the presence of the emotional stimuli. Canli et al [58] indicated that they chose women because “they report more intense emotional experiences, and show more physiological reactivity in concordance with valence judgments than men”. Most of the studies we have aforementioned have been conducted with female subjects [5,6,9,11]. Scharmüller et al [11] pointed out that they restricted their study to use only female subjects since the prevalence of spider phobia is higher in them. Moreover, Schienle et al [6] remarked that most of the spiders’ phobia sufferers were females. Another limitation could be the absence of control subjects to compare with, which could constitute a future extension of the current work.

### Conclusions

In conclusion, the results that we have obtained with VR is similar to the results obtained using real stimuli, in terms of fMRI brain activations. In fact, the main activations we found in the occipital and frontal areas are consistent with those found in previous studies that were conducted with spider phobic subjects using pictures or videos of real animals as stimuli. Moreover, the activation in the cuneus could be related to the sense of presence elicited in the subjects because of the navigation through the virtual environment. This finding opens the door to deeper investigations into the phobias, due to the fact that VR allows the recreation of normal life scenes in a more realistic and interactive way, that are impossible to achieve with other techniques. This kind of situation could allow, for example, the study of subjects with a mild phobia, whose fear cannot be excited by only the use of photographs.

Once the validity of the use of VR as a stimulus for evoking the phobia is evaluated, the next step will be to prove the usefulness of VR during the assessment of the brain activations after the subjects have passed through a psychological treatment to cure the disorder. This will be evaluated in future studies.

## Multimedia Appendix 1

PowerPoint of the presentation done during the REHAB 2013 Workshop, held in Venice (Italy) on the 5th of May 2013.

## Abbreviations

AC-PC line: anterior commissure – posterior commissure line
CBT: cognitive behavioral therapy
DSM: Diagnostic and Statistical Manual of Mental Disorders
DSM-IV: Diagnostic and Statistical Manual of Mental Disorders - 4th edition
EEG: electroencephalography
FA: flip angle
fMRI: functional magnetic resonance imaging
FWHM: full width at half maximum
MNI: Montreal Neurological Institute
MR: magnetic resonance
MRI: magnetic resonance imaging
PET: positron emission tomography
SPM: statistical parametric mapping
SPM8: statistical parametric mapping software version 8
T1: relaxation time
T2*: relaxation time
TE: echo time
TR: repetition time
VR: virtual reality
