# Supplementary material for: A Functional Magnetic Resonance Imaging Assessment of Small Animals’ Phobia Using Virtual Reality as a Stimulus
Source: JMIR Serious Games. 2014 Jun 27;2(1):e6. doi: 10.2196/games.2836 (PMC4307824; doi:10.2196/games.2836)
Supplement: Supplementary file 1 [file games_v2i1e6_app1.ppt]

## Slide 1
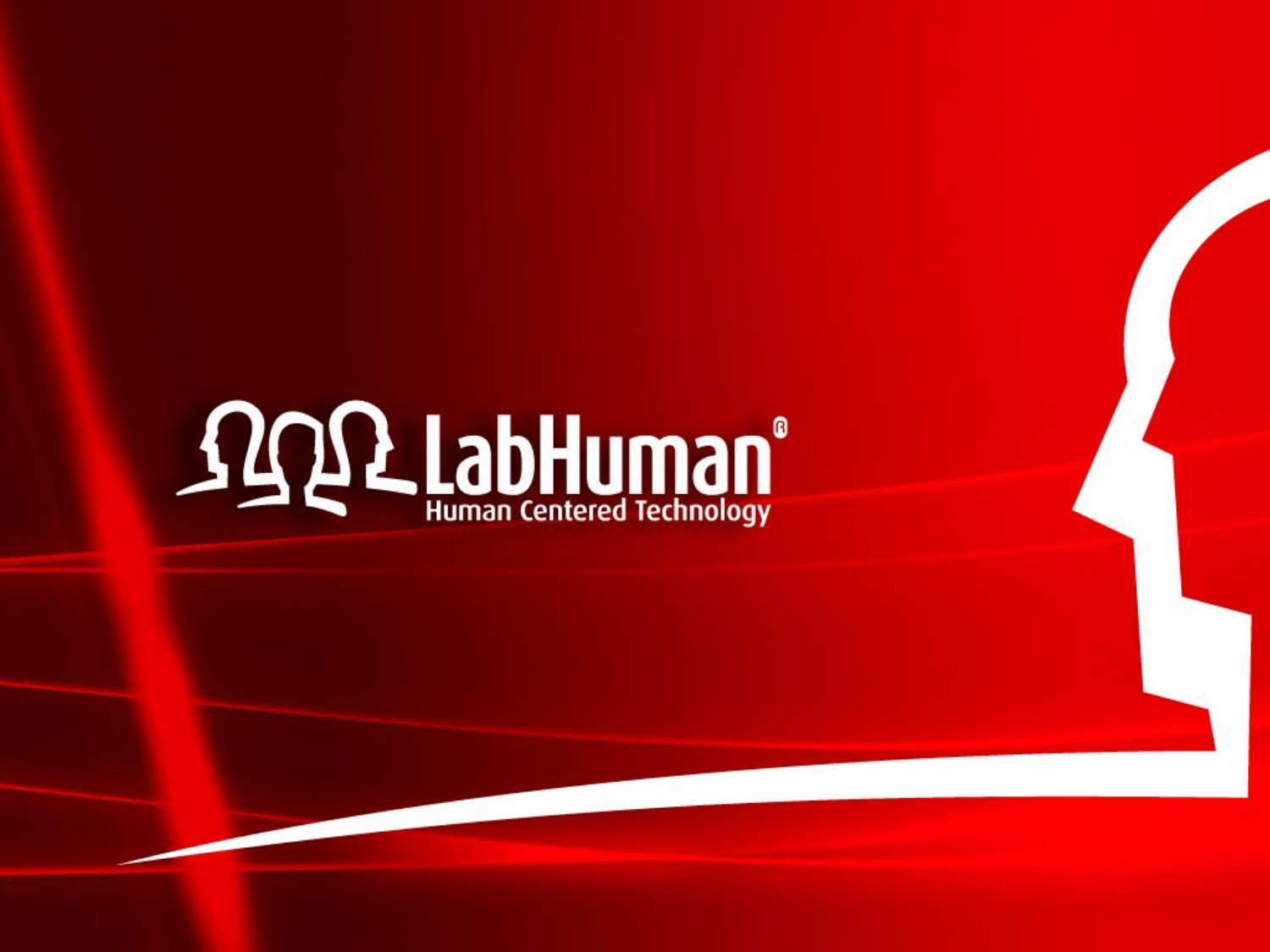

## Slide 2
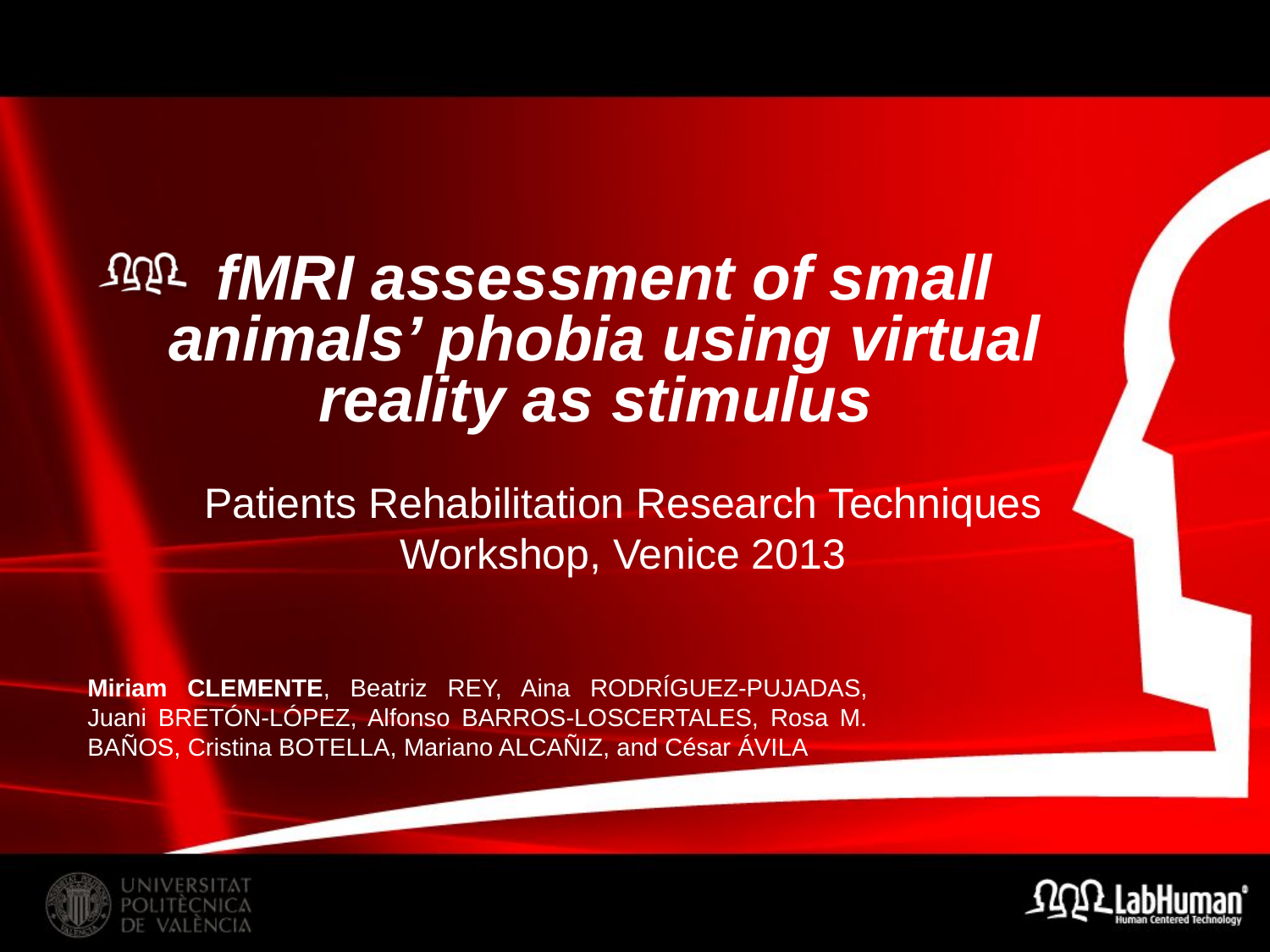

# fMRI assessment of small animals’ phobia using virtual reality as stimulus
Patients Rehabilitation Research Techniques Workshop, Venice 2013
Miriam CLEMENTE, Beatriz REY, Aina RODRÍGUEZ-PUJADAS, Juani BRETÓN-LÓPEZ, Alfonso BARROS-LOSCERTALES, Rosa M. BAÑOS, Cristina BOTELLA, Mariano ALCAÑIZ, and César ÁVILA

## Slide 3
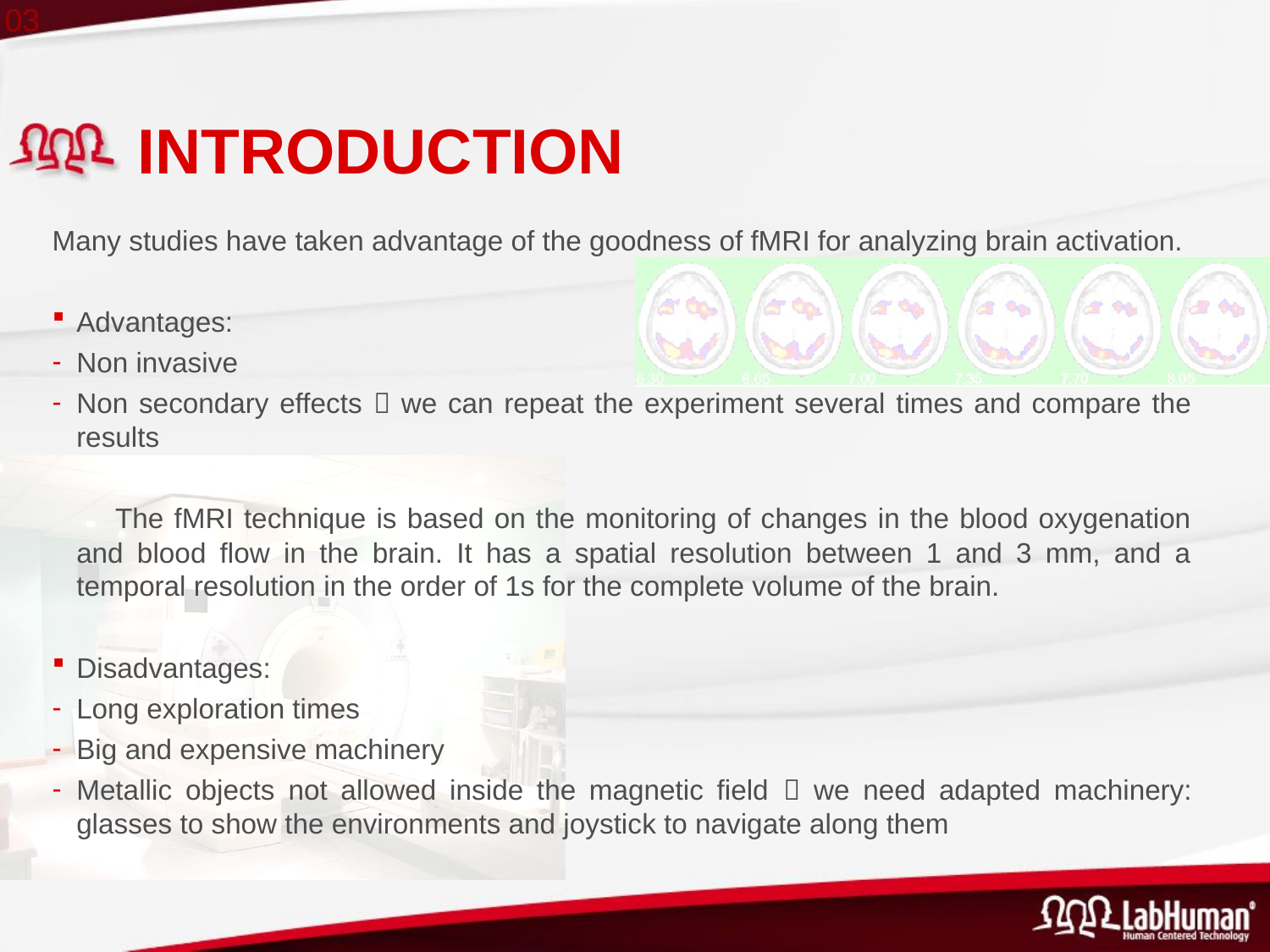

0<number>
# INTRODUCTION
Many studies have taken advantage of the goodness of fMRI for analyzing brain activation.
Advantages:
Non invasive
Non secondary effects  we can repeat the experiment several times and compare the results
 	 The fMRI technique is based on the monitoring of changes in the blood oxygenation and blood flow in the brain. It has a spatial resolution between 1 and 3 mm, and a temporal resolution in the order of 1s for the complete volume of the brain.
Disadvantages:
Long exploration times
Big and expensive machinery
Metallic objects not allowed inside the magnetic field  we need adapted machinery: glasses to show the environments and joystick to navigate along them

## Slide 4
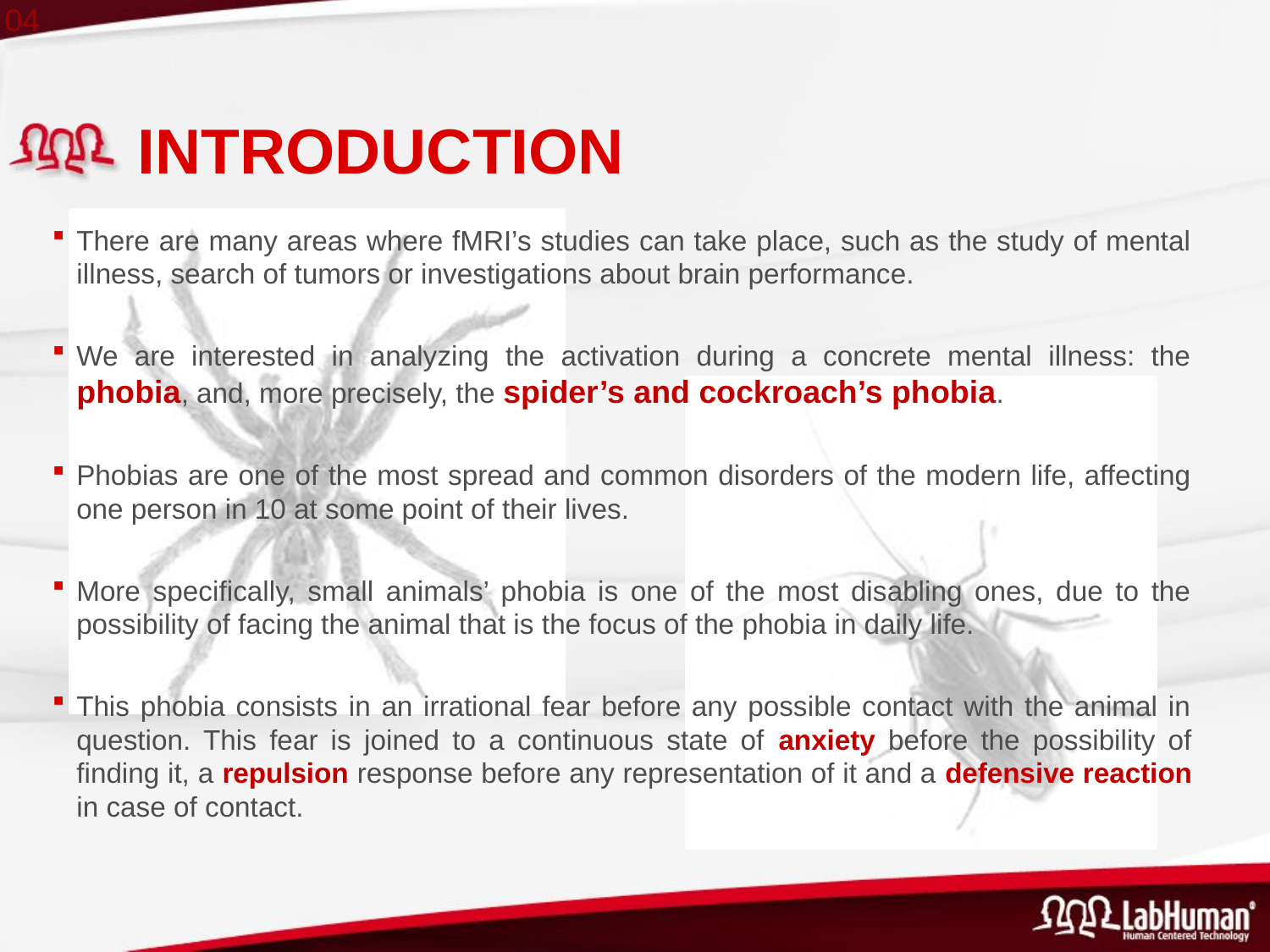

0<number>
# INTRODUCTION
There are many areas where fMRI’s studies can take place, such as the study of mental illness, search of tumors or investigations about brain performance.
We are interested in analyzing the activation during a concrete mental illness: the phobia, and, more precisely, the spider’s and cockroach’s phobia.
Phobias are one of the most spread and common disorders of the modern life, affecting one person in 10 at some point of their lives.
More specifically, small animals’ phobia is one of the most disabling ones, due to the possibility of facing the animal that is the focus of the phobia in daily life.
This phobia consists in an irrational fear before any possible contact with the animal in question. This fear is joined to a continuous state of anxiety before the possibility of finding it, a repulsion response before any representation of it and a defensive reaction in case of contact.

## Slide 5
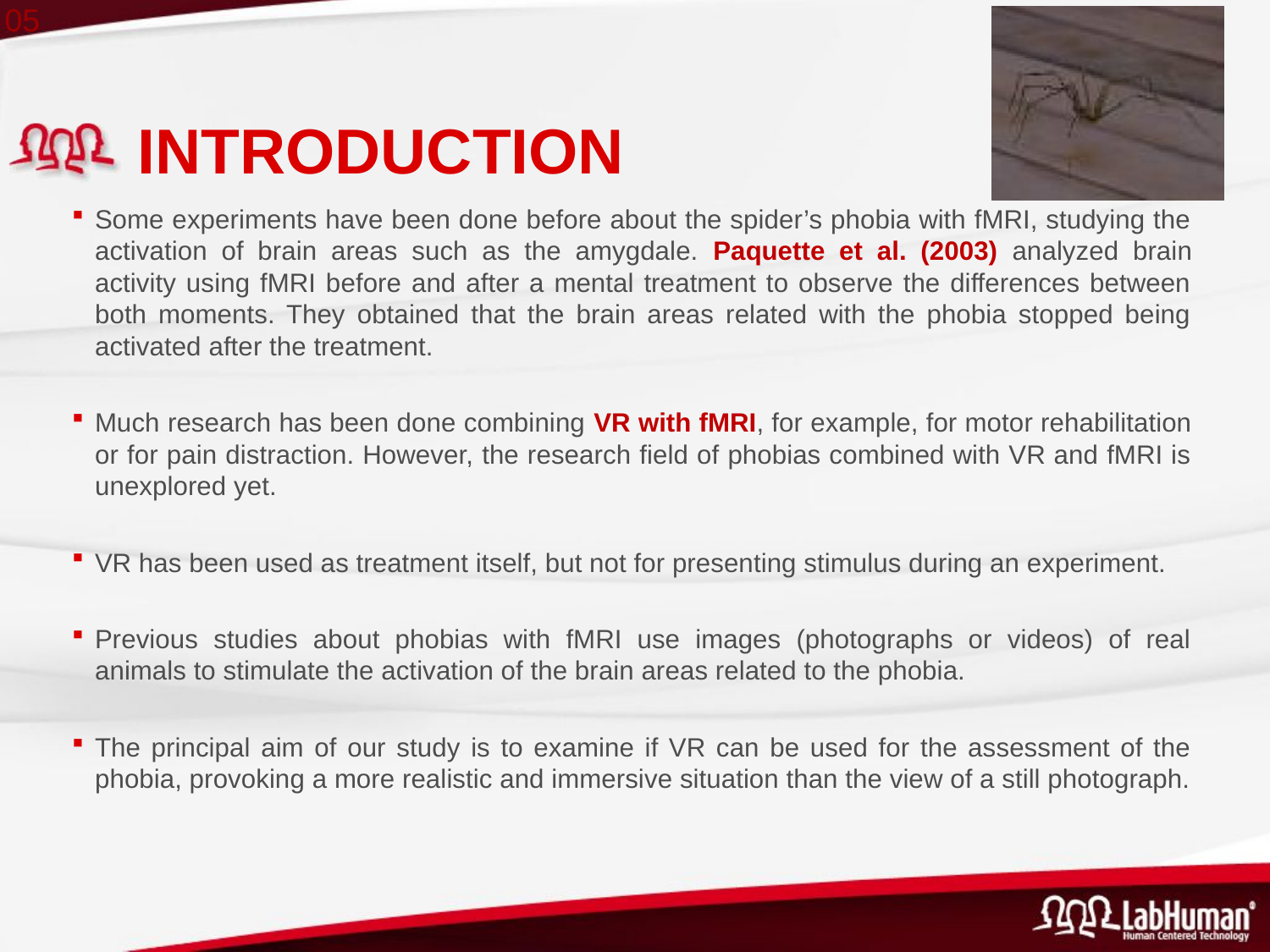

0<number>
# INTRODUCTION
Some experiments have been done before about the spider’s phobia with fMRI, studying the activation of brain areas such as the amygdale. Paquette et al. (2003) analyzed brain activity using fMRI before and after a mental treatment to observe the differences between both moments. They obtained that the brain areas related with the phobia stopped being activated after the treatment.
Much research has been done combining VR with fMRI, for example, for motor rehabilitation or for pain distraction. However, the research field of phobias combined with VR and fMRI is unexplored yet.
VR has been used as treatment itself, but not for presenting stimulus during an experiment.
Previous studies about phobias with fMRI use images (photographs or videos) of real animals to stimulate the activation of the brain areas related to the phobia.
The principal aim of our study is to examine if VR can be used for the assessment of the phobia, provoking a more realistic and immersive situation than the view of a still photograph.

## Slide 6
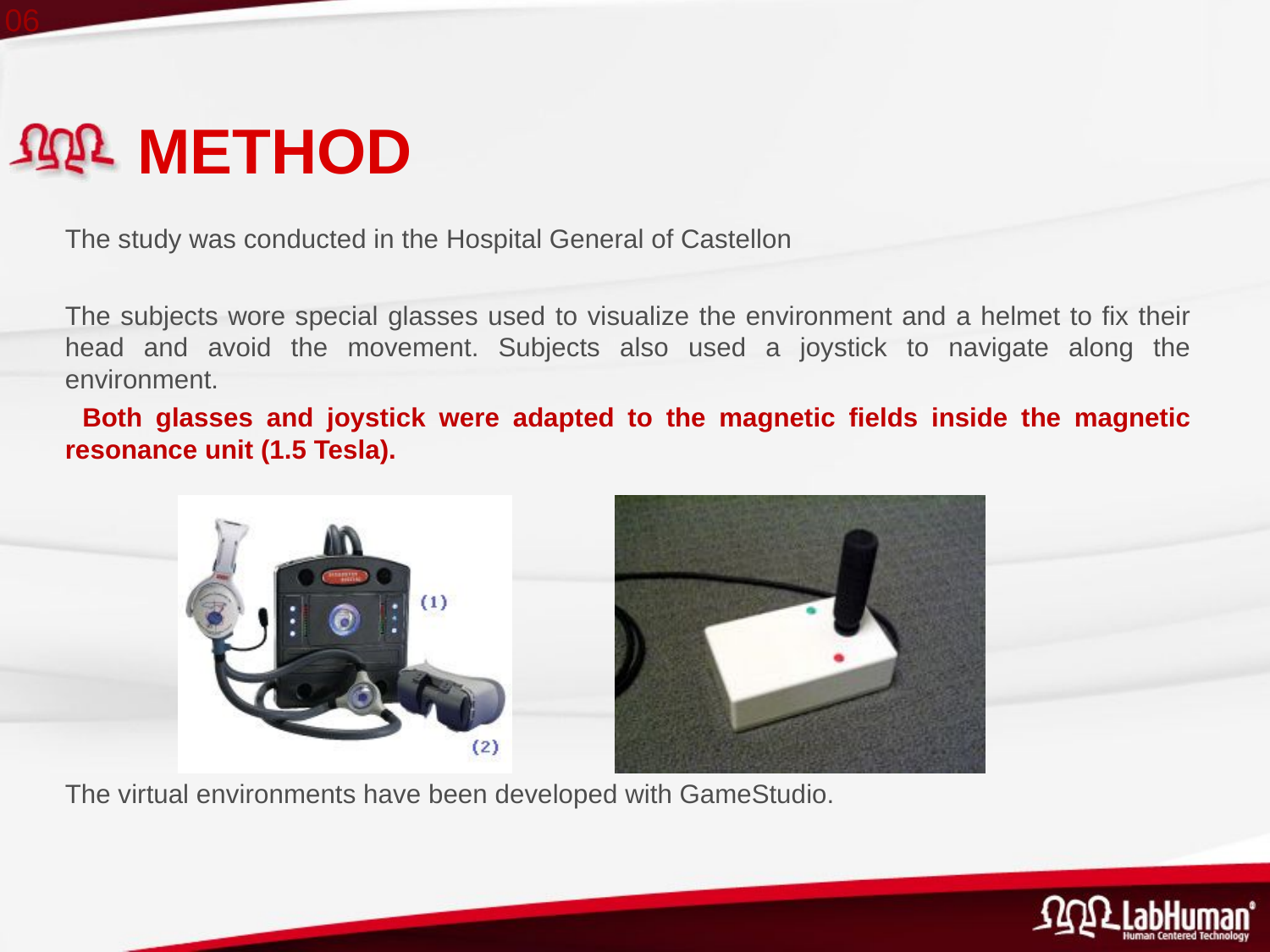

0<number>
# METHOD
 	The study was conducted in the Hospital General of Castellon
	The subjects wore special glasses used to visualize the environment and a helmet to fix their head and avoid the movement. Subjects also used a joystick to navigate along the environment.
 Both glasses and joystick were adapted to the magnetic fields inside the magnetic resonance unit (1.5 Tesla).
	The virtual environments have been developed with GameStudio.

## Slide 7
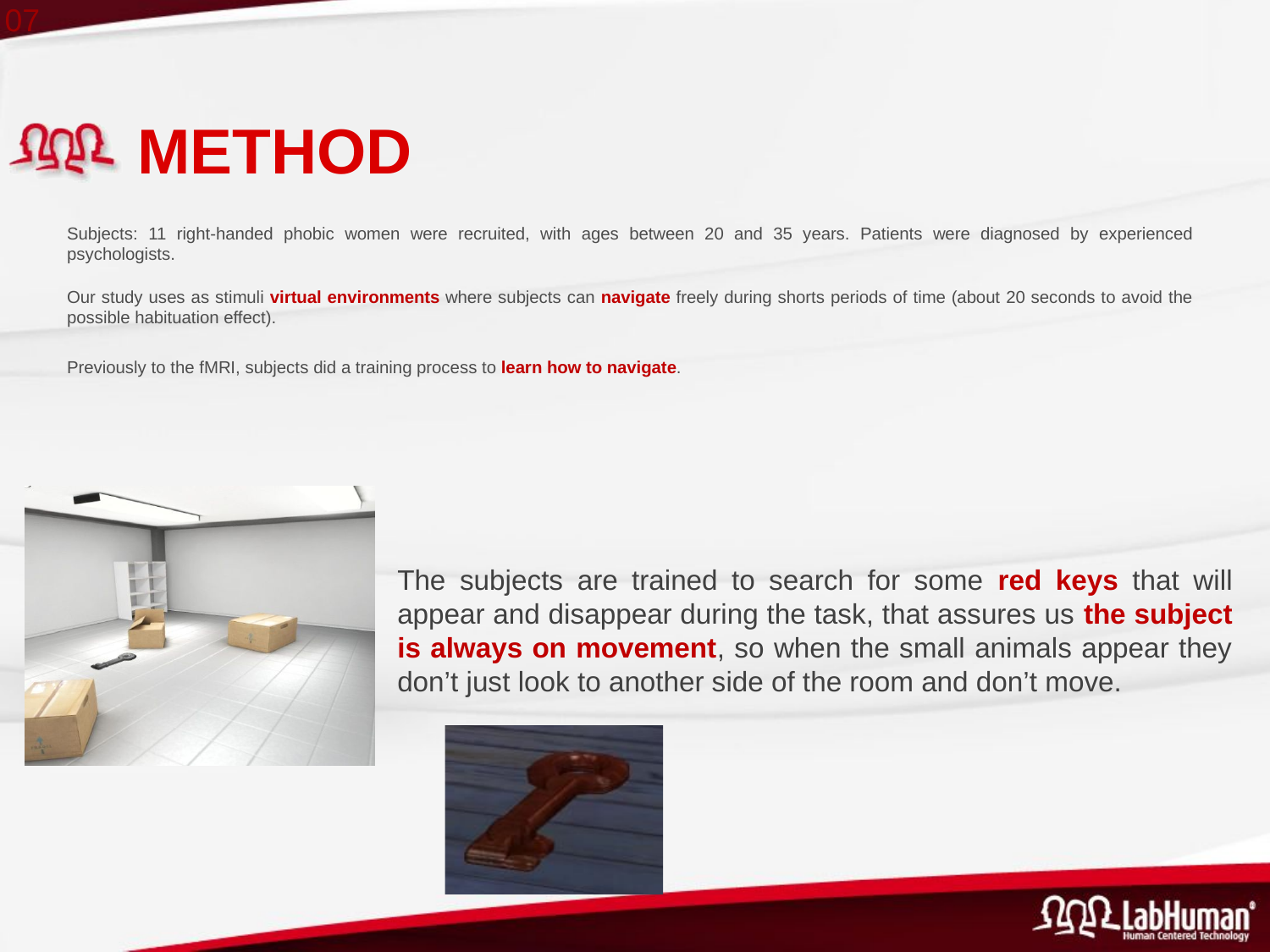

0<number>
# METHOD
	Subjects: 11 right-handed phobic women were recruited, with ages between 20 and 35 years. Patients were diagnosed by experienced psychologists.
	Our study uses as stimuli virtual environments where subjects can navigate freely during shorts periods of time (about 20 seconds to avoid the possible habituation effect).
	Previously to the fMRI, subjects did a training process to learn how to navigate.
The subjects are trained to search for some red keys that will appear and disappear during the task, that assures us the subject is always on movement, so when the small animals appear they don’t just look to another side of the room and don’t move.

## Slide 8
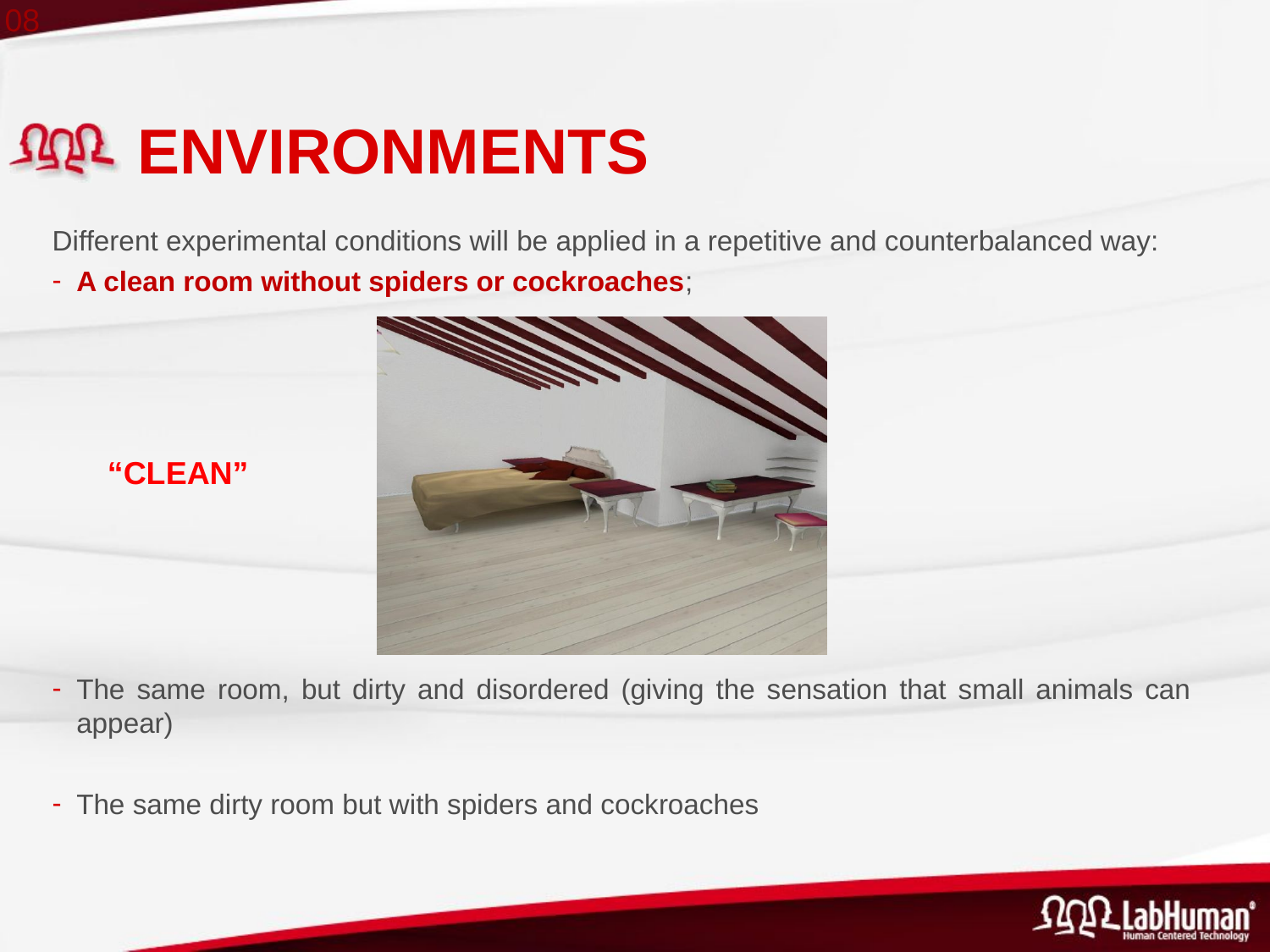

0<number>
# ENVIRONMENTS
Different experimental conditions will be applied in a repetitive and counterbalanced way:
A clean room without spiders or cockroaches;
The same room, but dirty and disordered (giving the sensation that small animals can appear)
The same dirty room but with spiders and cockroaches
“CLEAN”

## Slide 9
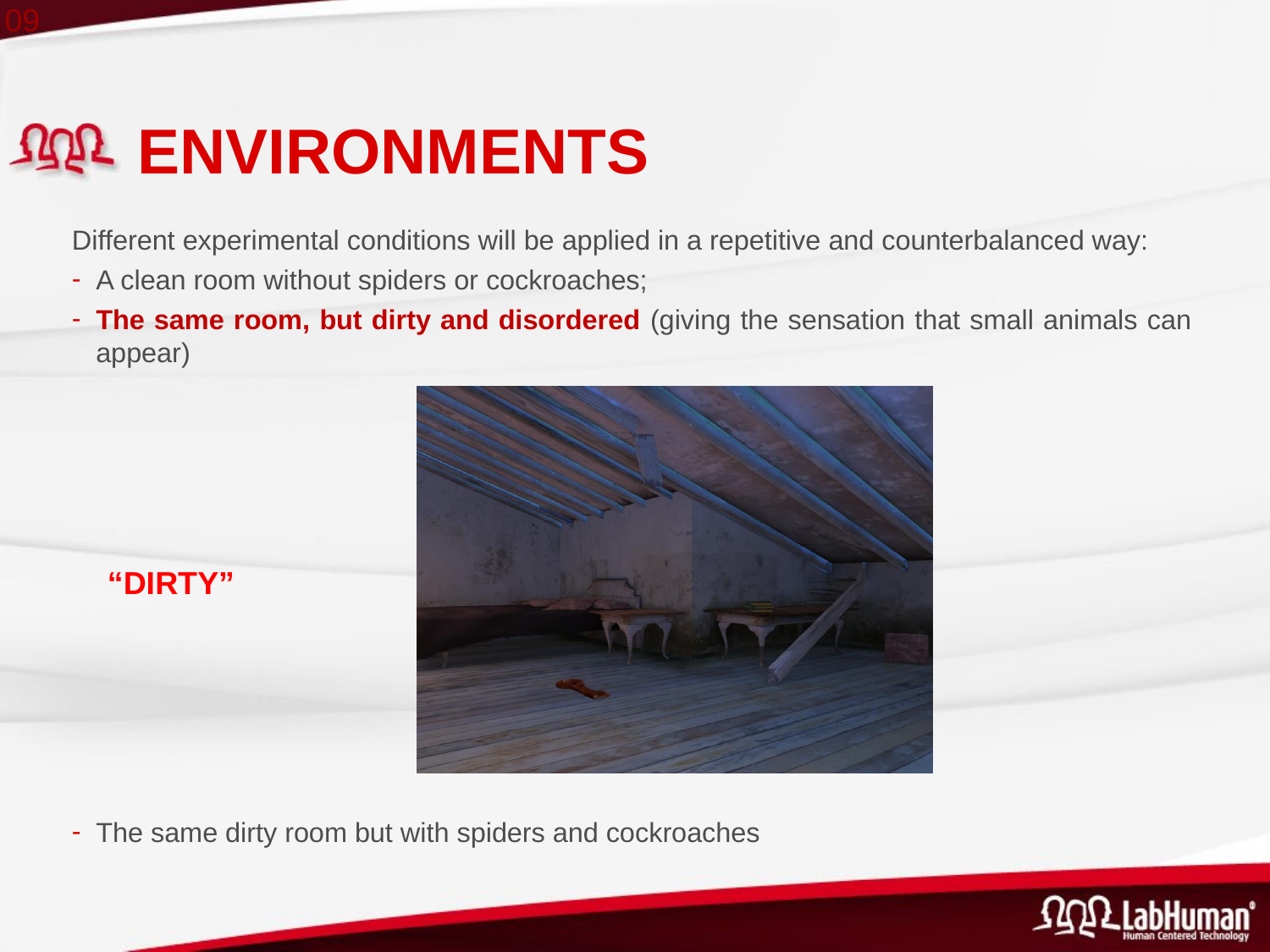

0<number>
# ENVIRONMENTS
Different experimental conditions will be applied in a repetitive and counterbalanced way:
A clean room without spiders or cockroaches;
The same room, but dirty and disordered (giving the sensation that small animals can appear)
The same dirty room but with spiders and cockroaches
“DIRTY”

## Slide 10
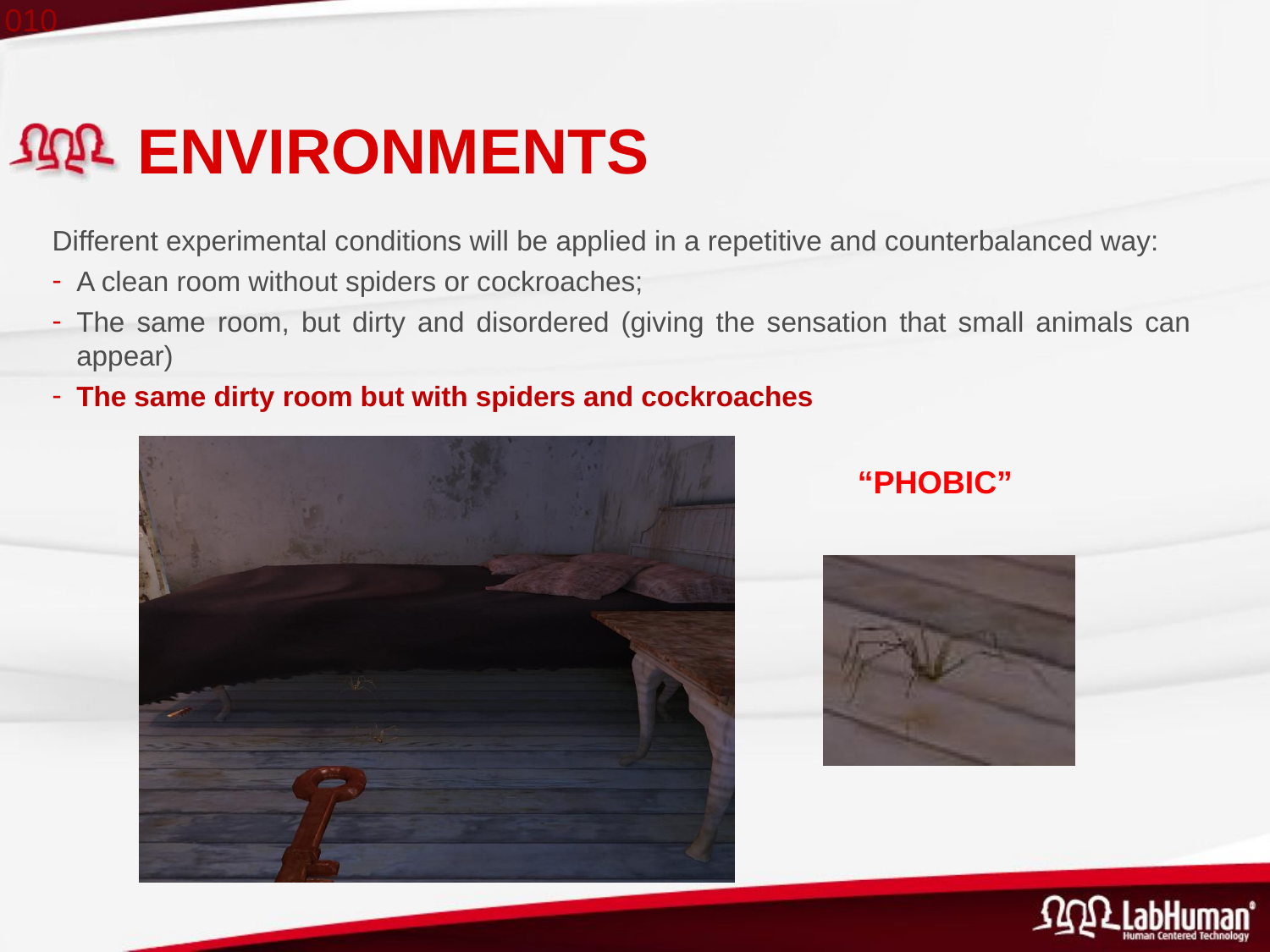

0<number>
# ENVIRONMENTS
Different experimental conditions will be applied in a repetitive and counterbalanced way:
A clean room without spiders or cockroaches;
The same room, but dirty and disordered (giving the sensation that small animals can appear)
The same dirty room but with spiders and cockroaches
“PHOBIC”

## Slide 11
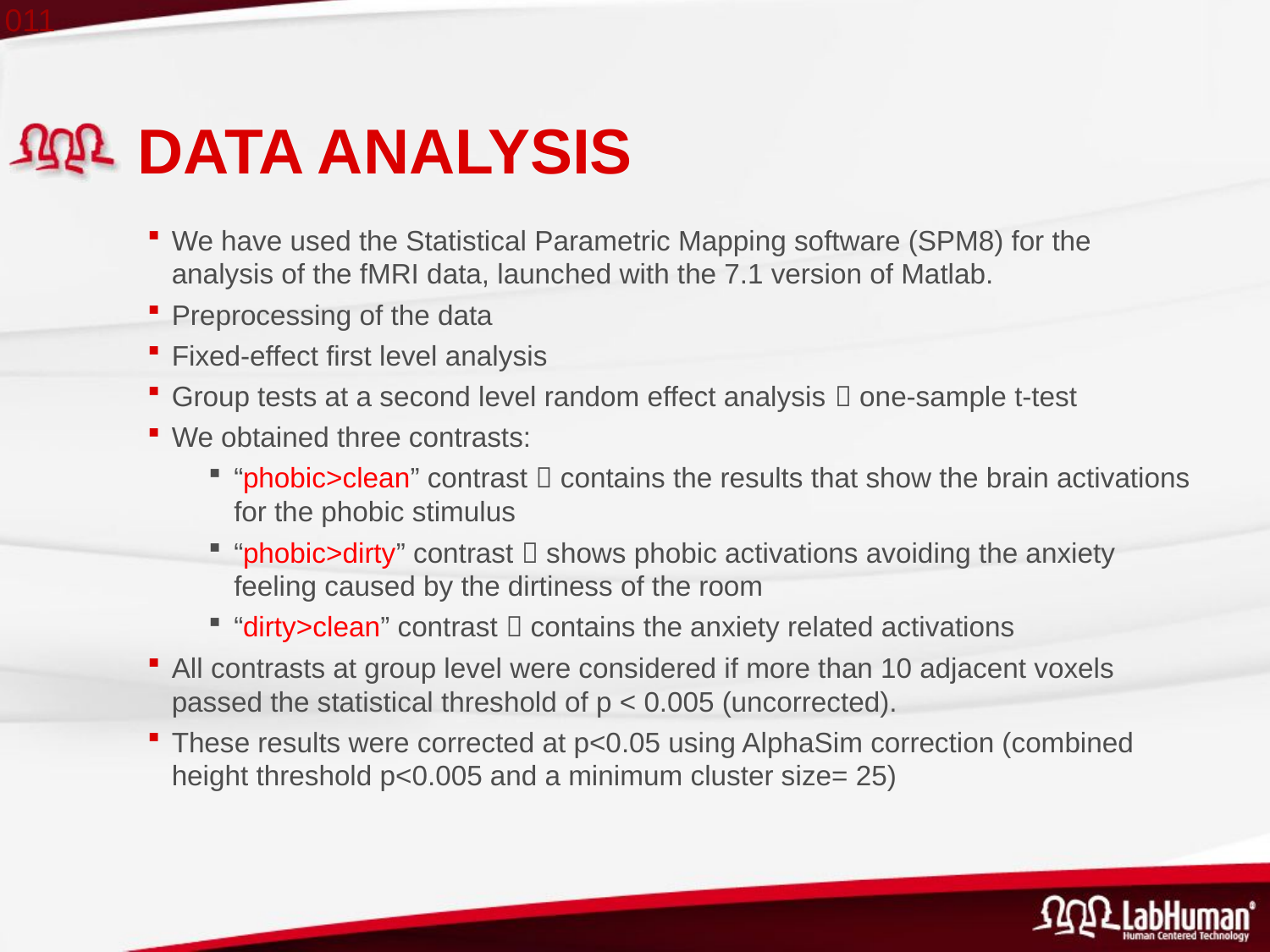

0<number>
# DATA ANALYSIS
We have used the Statistical Parametric Mapping software (SPM8) for the analysis of the fMRI data, launched with the 7.1 version of Matlab.
Preprocessing of the data
Fixed-effect first level analysis
Group tests at a second level random effect analysis  one-sample t-test
We obtained three contrasts:
“phobic>clean” contrast  contains the results that show the brain activations for the phobic stimulus
“phobic>dirty” contrast  shows phobic activations avoiding the anxiety feeling caused by the dirtiness of the room
“dirty>clean” contrast  contains the anxiety related activations
All contrasts at group level were considered if more than 10 adjacent voxels passed the statistical threshold of p < 0.005 (uncorrected).
These results were corrected at p<0.05 using AlphaSim correction (combined height threshold p<0.005 and a minimum cluster size= 25)

## Slide 12
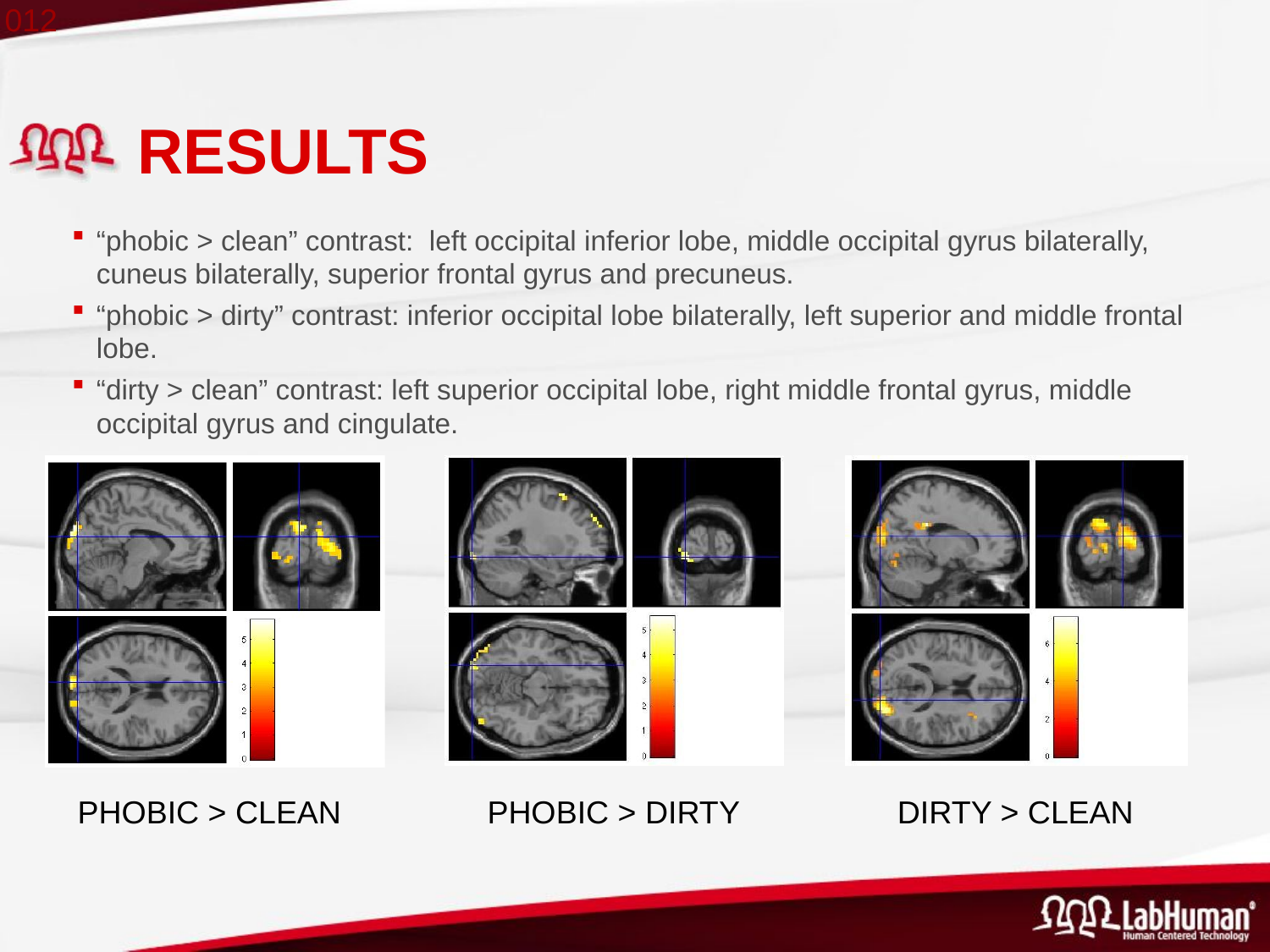

0<number>
# RESULTS
“phobic > clean” contrast: left occipital inferior lobe, middle occipital gyrus bilaterally, cuneus bilaterally, superior frontal gyrus and precuneus.
“phobic > dirty” contrast: inferior occipital lobe bilaterally, left superior and middle frontal lobe.
“dirty > clean” contrast: left superior occipital lobe, right middle frontal gyrus, middle occipital gyrus and cingulate.
PHOBIC > CLEAN
PHOBIC > DIRTY
DIRTY > CLEAN

## Slide 13
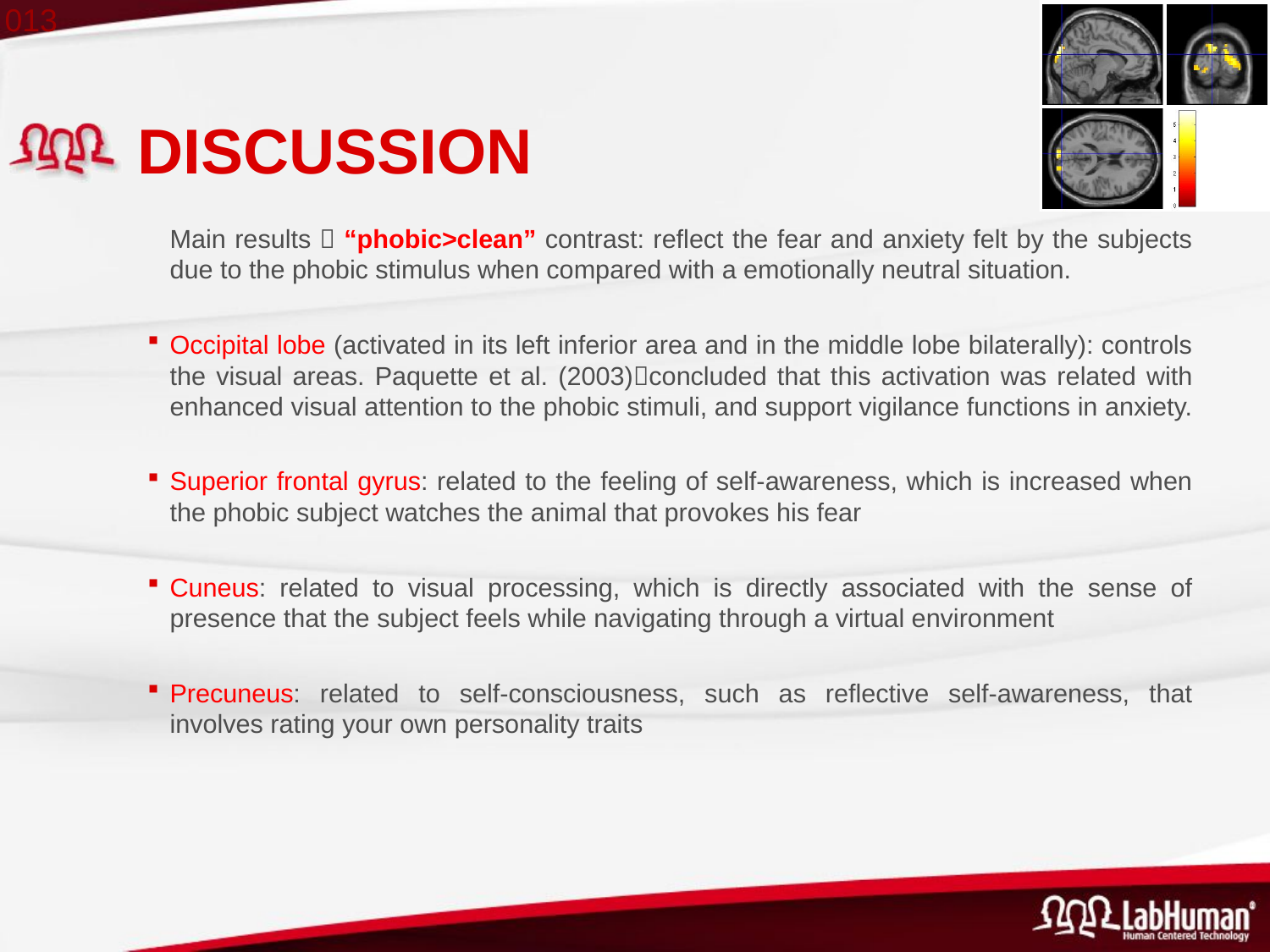

0<number>
# DISCUSSION
	Main results  “phobic>clean” contrast: reflect the fear and anxiety felt by the subjects due to the phobic stimulus when compared with a emotionally neutral situation.
Occipital lobe (activated in its left inferior area and in the middle lobe bilaterally): controls the visual areas. Paquette et al. (2003)concluded that this activation was related with enhanced visual attention to the phobic stimuli, and support vigilance functions in anxiety.
Superior frontal gyrus: related to the feeling of self-awareness, which is increased when the phobic subject watches the animal that provokes his fear
Cuneus: related to visual processing, which is directly associated with the sense of presence that the subject feels while navigating through a virtual environment
Precuneus: related to self-consciousness, such as reflective self-awareness, that involves rating your own personality traits

## Slide 14
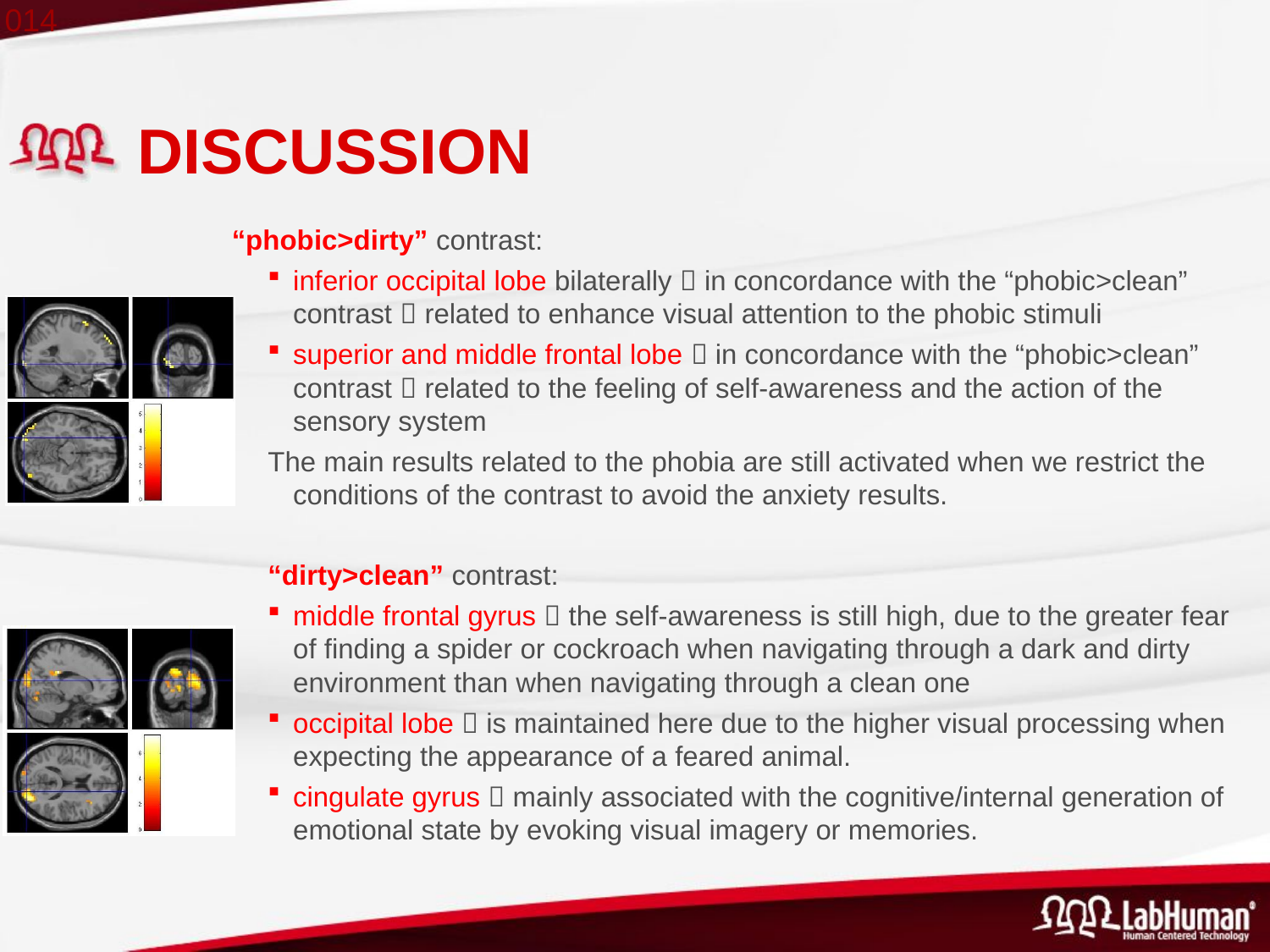

0<number>
# DISCUSSION
	“phobic>dirty” contrast:
inferior occipital lobe bilaterally  in concordance with the “phobic>clean” contrast  related to enhance visual attention to the phobic stimuli
superior and middle frontal lobe  in concordance with the “phobic>clean” contrast  related to the feeling of self-awareness and the action of the sensory system
The main results related to the phobia are still activated when we restrict the conditions of the contrast to avoid the anxiety results.
“dirty>clean” contrast:
middle frontal gyrus  the self-awareness is still high, due to the greater fear of finding a spider or cockroach when navigating through a dark and dirty environment than when navigating through a clean one
occipital lobe  is maintained here due to the higher visual processing when expecting the appearance of a feared animal.
cingulate gyrus  mainly associated with the cognitive/internal generation of emotional state by evoking visual imagery or memories.

## Slide 15
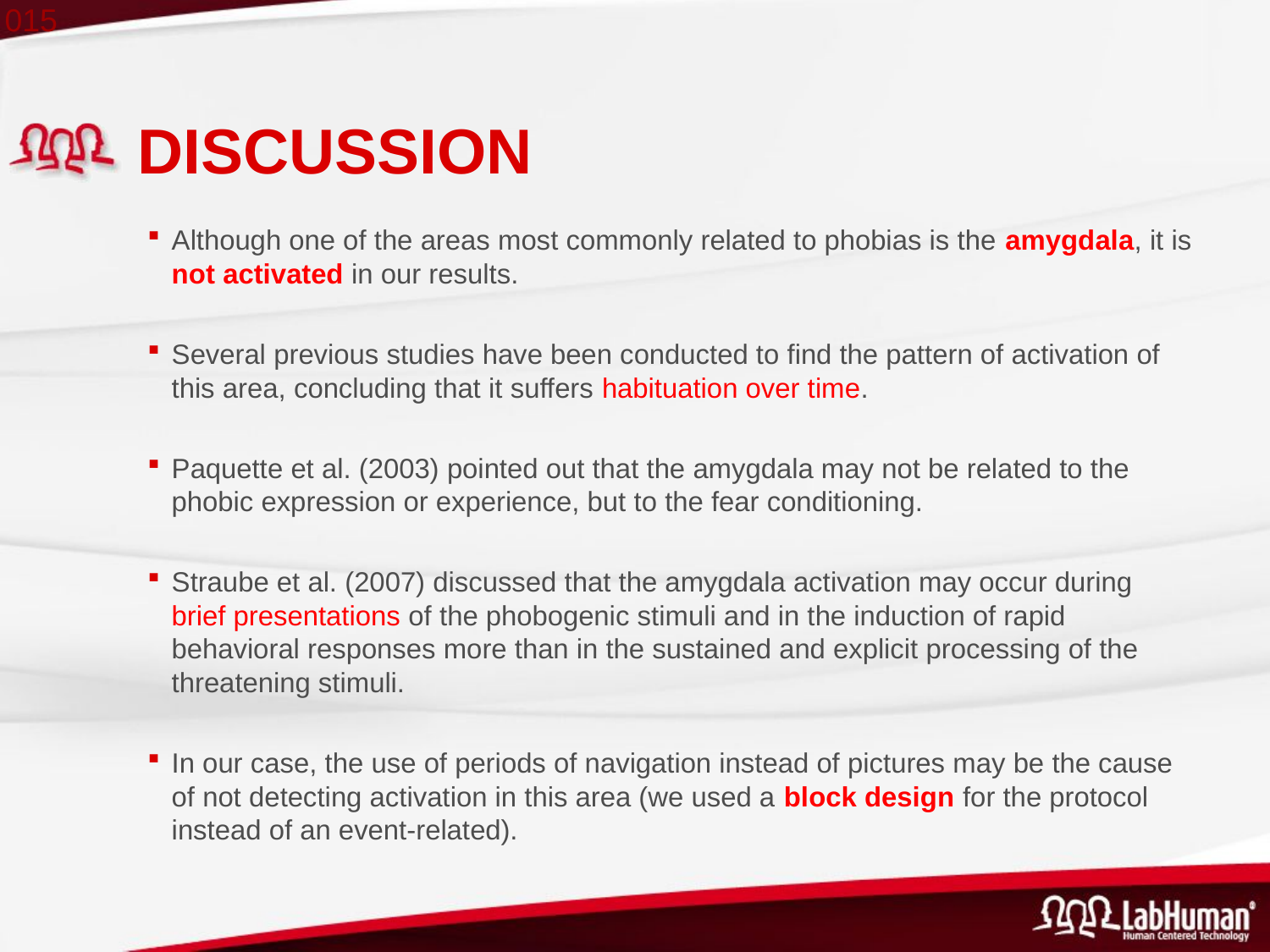

0<number>
# DISCUSSION
Although one of the areas most commonly related to phobias is the amygdala, it is not activated in our results.
Several previous studies have been conducted to find the pattern of activation of this area, concluding that it suffers habituation over time.
Paquette et al. (2003) pointed out that the amygdala may not be related to the phobic expression or experience, but to the fear conditioning.
Straube et al. (2007) discussed that the amygdala activation may occur during brief presentations of the phobogenic stimuli and in the induction of rapid behavioral responses more than in the sustained and explicit processing of the threatening stimuli.
In our case, the use of periods of navigation instead of pictures may be the cause of not detecting activation in this area (we used a block design for the protocol instead of an event-related).

## Slide 16
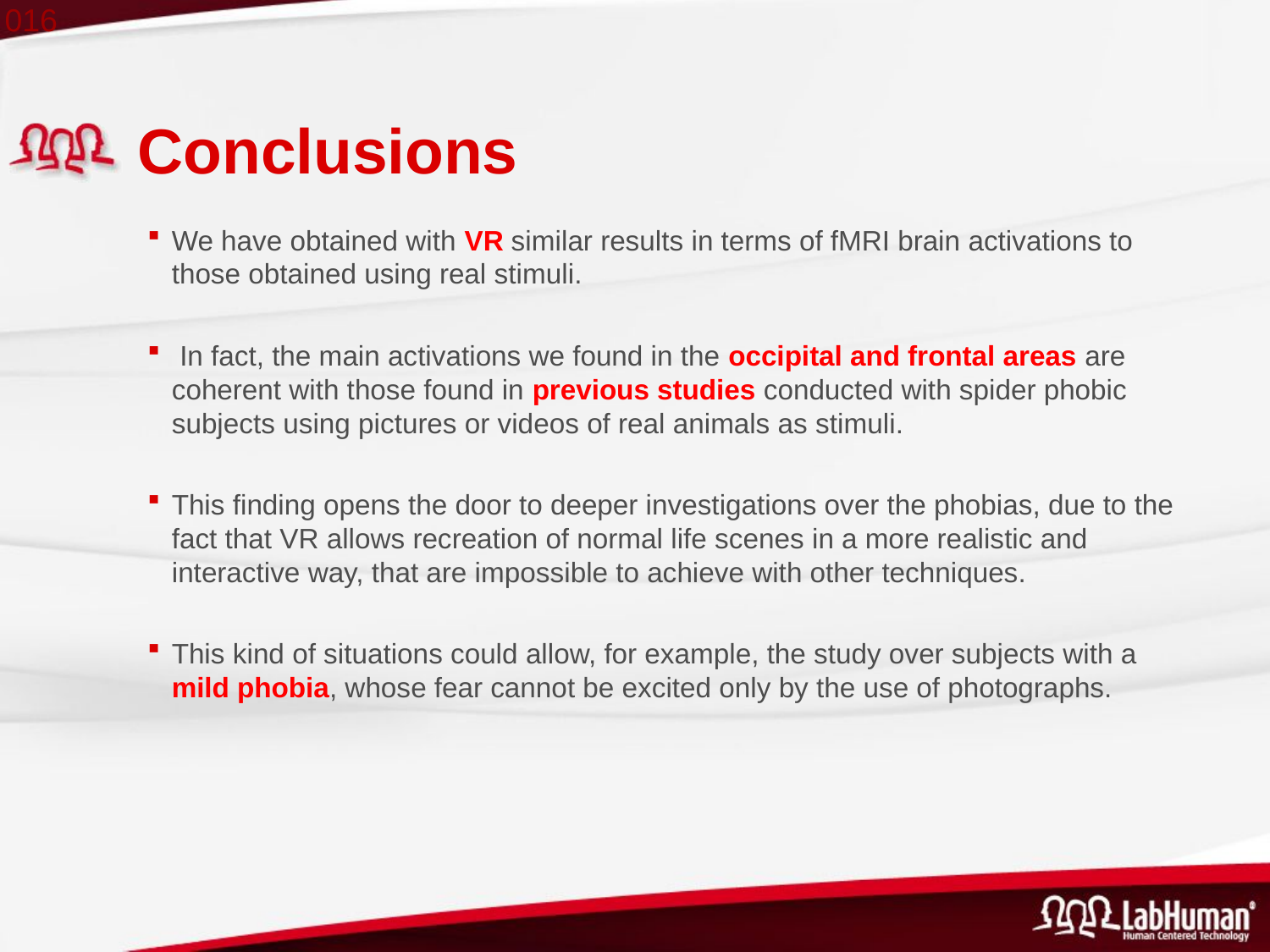

0<number>
# Conclusions
We have obtained with VR similar results in terms of fMRI brain activations to those obtained using real stimuli.
 In fact, the main activations we found in the occipital and frontal areas are coherent with those found in previous studies conducted with spider phobic subjects using pictures or videos of real animals as stimuli.
This finding opens the door to deeper investigations over the phobias, due to the fact that VR allows recreation of normal life scenes in a more realistic and interactive way, that are impossible to achieve with other techniques.
This kind of situations could allow, for example, the study over subjects with a mild phobia, whose fear cannot be excited only by the use of photographs.

## Slide 17
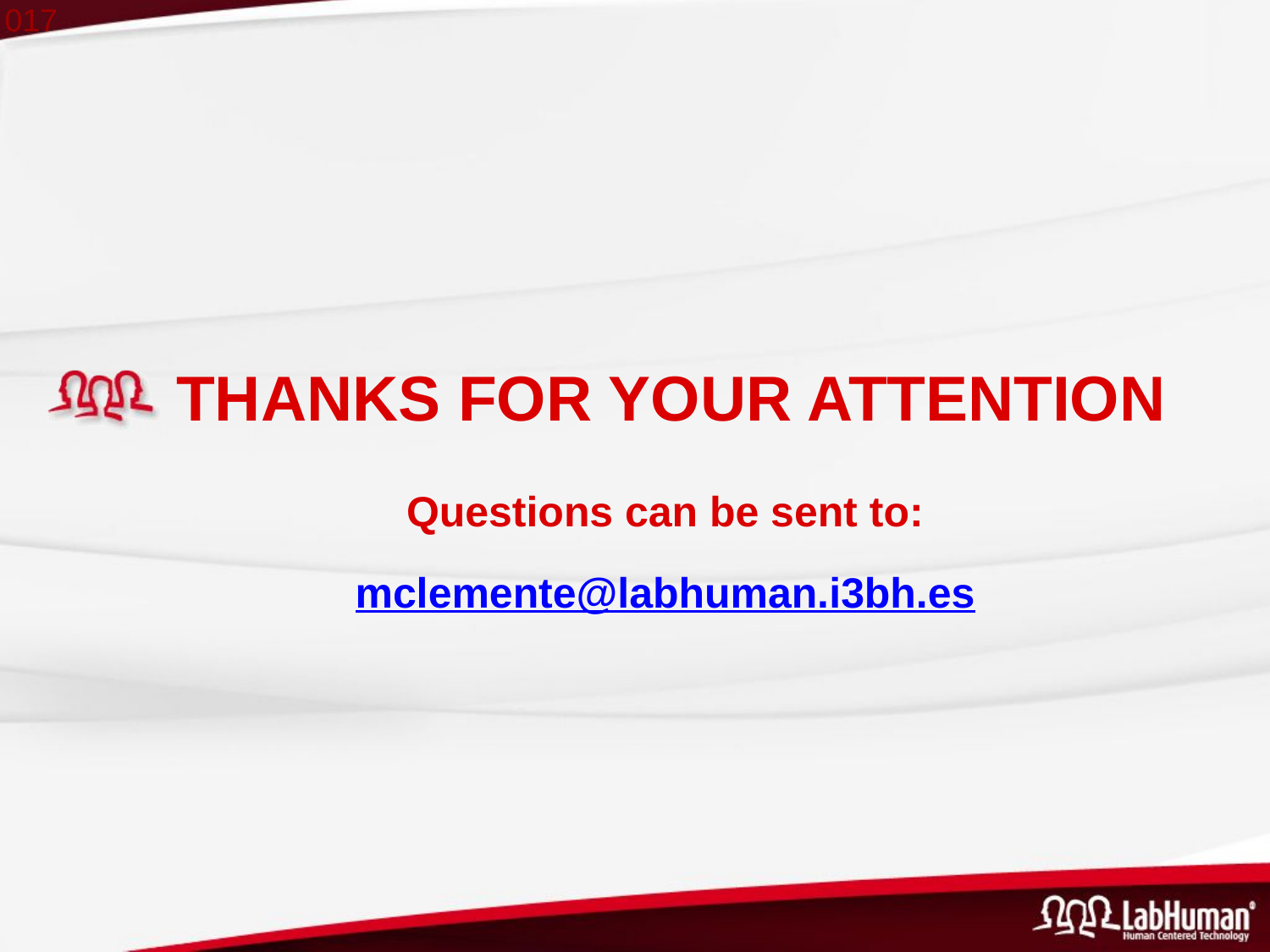

0<number>
# THANKS FOR YOUR ATTENTION Questions can be sent to: mclemente@labhuman.i3bh.es
